# 1D228 Attenuates Sorafenib Resistance in Renal Cell Carcinoma Models by Dual Targeting c-Met and AXL

**DOI:** 10.3390/cells15161450

**Published:** 2026-08-12

**Authors:** Hanxu Qian, Huimin Ren, Lei Xu, Xingge Hu, Yihong Sun, Qing Ju, Chenguo Zhang, Shuo Liu, Baijiao An, Chunhua Yang, Xingjie Liu, Yin Zhang

**Affiliations:** 1 Shandong Center of Technology Innovation for Molecular Targeting and Intelligent Diagnosis and Treatment, Shandong Key Lab of Complex Medical Intelligence and Aging, Shandong Medical and Pharmaceutical University, Yantai 264003, China; xuxu162022@163.com (H.Q.); hmren4796@stu.sdmpu.edu.cn (H.R.); 2023081002@stu.sdmpu.edu.cn (L.X.); 2025021033@stu.sdmpu.edu.cn (X.H.); 2023081020@stu.sdmpu.edu.cn (Y.S.); 2024071024@stu.sdmpu.edu.cn (Q.J.); 244230102@stu.sdmpu.edu.cn (C.Z.); 2025072003@stu.sdmpu.edu.cn (S.L.); anbj3@bzmc.edu.cn (B.A.); yangchunhua@bzmc.edu.cn (C.Y.); 2 Medicine and Pharmacy Research Center, Shandong Medical and Pharmaceutical University, Yantai 264003, China; 3 Department of Oncology, Shandong Medical and Pharmaceutical University Hospital, 522 Huanghe 3rd Road, Binzhou 256603, China

**Keywords:** renal cell carcinoma, sorafenib resistance, c-Met, AXL, 1D228

## Abstract

Sorafenib is widely used to treat metastatic renal cell carcinoma (RCC); however, the acquired drug resistance limits its efficacy and application in clinical practice. The receptor tyrosine kinases c-Met and AXL play important roles in cancer progression and are involved in tyrosine kinase inhibitor-induced drug resistance in cancers, but whether these two receptors also contribute to sorafenib-induced drug resistance in RCC is unclear. In this study, we evaluated our synthesized compound 1D228, a TKI derived from Tepotinib, in sorafenib-resistant RCC models, which demonstrated further inhibition in sorafenib-resistant RCC cells, and induced 25% more reduction in resistant RCC tumor size by 1D228 combined with sorafenib compared with sorafenib monotherapy in animal models. Mechanistically, resistant RCC exhibited elevated phosphorylation of c-Met and AXL, which was effectively suppressed by 1D228. These findings indicated that compound 1D228 sensitized the sorafenib resistance of RCC by dual targeting the c-Met and AXL signaling pathways. This study suggests that 1D228 may represent a promising preclinical therapeutic strategy for RCC patients with sorafenib resistance mediated by c-Met and AXL activation.

## 1. Introduction

Although sorafenib is no longer recommended as the firstline therapy for renal cell carcinoma (RCC), it is still widely used due to its relatively low toxicity and easy management during treatment, as an alternative therapy after other TKI treatment (cabozantinib), and a cost-effective solution in low-income countries or territories [1,2,3,4,5,6,7]. Thus, sorafenib resistance remains a meaningful issue to be addressed in an effective way in the treatment of renal cell carcinoma. Mechanisms of sorafenib resistance include the alternative activated signaling pathway [8,9,10,11], inhibition of autophagy or ferroptosis, potential of tumor stem cells, non-canonical tumor angiogenesis, as well as the efflux activity [12,13,14,15,16,17,18,19,20,21]. Among these mechanisms, alternative activation of other signaling is important for cancer cell survival [22,23,24,25]. Thus, other inhibitors have been tested to resensitize sorafenib in cancer treatment [26,27,28,29,30]. For example, the mTOR inhibitor everolimus has been studied in animals and is under phase I/II trials for resistant HCC and RCC, but the liver and skin toxicity risk was increased [31,32]. Immune therapy, for example, PD-1 antibodies, may produce significant benefits for the RCC patients, however, the high cost is not affordable for most of the patients [5,33,34,35]. Despite multiple therapeutic combinations being proposed for sorafenib resistance, the toxicity risk and effectiveness are still challenging.

c-Met activation is one of the most studied mechanisms in TKI-induced resistance [31,36,37]. The strategy of cabozantinib treatment is the standard therapy for the second-line therapy for RCC, however, the toxicity is much higher due to it targeting multiple tyrosine kinases such as VEGFRs, RET, KIT, c-Met, AXL, etc. [38,39]. Lenvatinib is also recommended for second-line therapy together with the mTOR inhibitor, but this increases toxicity as it targets the vascular system [40,41,42]. Tepotinib or savolitinib targets c-Met, which showed significant inhibition of cancer cells [43,44,45]. But patients with AXL mutation or hyperactivation might not benefit from tepotinib and savolitinib as they cannot suppress AXL. AXL activation is also studied in sorafenib-resistant tumors, and treatment by specific AXL-targeting inhibitors or TKIs was applied in clinical trials or in the lab [46,47,48]. Although cabozantinib can block both c-Met and AXL, its systemic toxicity is still a big risk to be carefully managed in clinical practice. Either dose reductions or drug discontinuation would cause other issues in RCC patients. To date, a specific, less toxic, and highly cost-effective drug remains imperative to be developed for the treatment of sorafenib-induced drug resistance in RCC.

In our previous study, we synthesized a new compound 1D228, derived from tepotinib, which targets c-Met and AXL, as well as NTRK [49]. 1D228 exhibited pronounced inhibition on several tumors by targeting both cancer cells and endothelial cells. However, its potential for inhibition on RCC is not studied. Recent studies reported the importance of c-Met and AXL in TKI induced resistance, and targeting AXL may overcome c-Met-induced drug resistance [50,51,52]. Therefore, it is interesting to test if 1D228 could also overcome sorafenib-induced resistance in RCC.

In our current work, we found that 1D228 resensitized RCC to sorafenib at low doses. 1D228 inhibited cell proliferation and migration in both in vivo and in vitro models and restored the sensitivity of tumor blood vessels to sorafenib. Theoretically, our agent 1D228 selectively targets AXL and c-Met, offering potentially less toxicity compared with other multi-target TKIs, which might minimize the broad TKI-induced adverse effects. Compared with tepotinib, 1D228 might be more effective as it showed better inhibition of c-Met than tepotinib (demonstrated in the published paper [49]). Additionally, 1D228 targets both c-Met and AXL, which may produce better effects in inhibiting tumor growth. Our data suggested that 1D228 could serve as an alternative to tepotinib but also as a secondary therapy for sorafenib-resistant patients with c-Met or AXL activation.

## 2. Materials and Methods

### 2.1. Cell Culture and Transfection

Mouse renal cancer cell line RENCA (CL-0568) and human cell lines OSRC-2 (CL-0177), 786-O (CL-0010), CAKI-1 (CL-0052), A498 (CL-0254), ACHN (CL-0021), and 769P (CL-0009) were purchased from Procell Life Science & Technology Co (Wuhan, China). RENCA, OSRC-2, 786-O, and 769P cells were cultured in RPMI-1640 medium (MT10040C, Gibco, Grand Island, NY, USA). CAKI-1 cells were cultured in McCoy’s 5A medium (CM-0052, Procell, Wuhan, China). A498,and ACHN cells were cultured in MEM medium (CM-0254, Procell, Wuhan, China). These mediums are supplemented with 1% penicillin/streptomycin (P7630, Solarbio, Beijing, China) and 10% fetal bovine serum (FSS500, ExCell Bio, Taicang, China). Human clear cell renal cell carcinoma (ccRCC) cells were cultured in a humidified CO_2_ incubator (Thermo Fisher Scientific, Grand Island, NY, USA) maintained at 37 °C with 5% CO_2_. Post-thaw cells underwent two consecutive passages to adapt to the culture environment, and all experiments were carried out once cell proliferation became stable. All cell lines were mycoplasma-free prior to all experimental manipulations.

The pcDNA3.1-Flag-c-Met (NM_008591.3) and pcDNA3.1-Flag-AXL (NM_009465.4) plasmids were purchased from Weizhen Biotechnology (Jinan, China). siRNAs against c-Met and AXL were synthesized by Tsingke Biotech (Beijing, China). The siRNA sequences used were as follows: c-Met siRNA: 5′-GAACAGCGAGCUAAAUAUA-3′; AXL siRNA:5′-CCACUCAGAUGCUAGUGAA-3′. The negative control siRNA sequences used were as follows: sense strand: 5′-UUCUCCGAACGUGUCACGUTT-3′; antisense strand: 5′-ACGUGACACGUUCGGAGAA-3′. The cells were transfected with JetPRIME (Polyplus, 201000003, Illkirch, France) when they were 70 to 80% confluent and according to the manufacturer’s protocol. The transfection concentration of siRNA plasmids was 100 nM, while the dosage of overexpression plasmids for transfection was 2.5 μg/mL. The cells were harvested at 48–72 h post transfection for future experiments.

For drug treatment, 1D228 and sorafenib were dissolved in DMSO to prepare a stock solution at a concentration of 5 mM. The working solution was diluted using a culture medium for treatment.

### 2.2. Generation of Sorafenib-Resistant RENCA Cells

The sorafenib-resistant subline of RENCA cells (RENCA^R^) was established by a stepwise dose-escalation method. Parental RENCA cells were cultured in a complete medium containing sorafenib (MCE, HY-10201, Shanghai, China) starting at an initial concentration of 5 μM. The concentration of sorafenib gradually increased to 12 μM over 6 months until cells reached a stable proliferation status.

In this study, the RENCA^R^ cells were used within passage 15 after recovery from liquid nitrogen. Cell subculturing was strictly followed by ATCC and standard operating procedures. Cells were passaged when they reached the logarithmic growth phase at 70–80% confluence to ensure optimal cell status. The resistant cells were maintained in the culture medium containing 7 μM sorafenib. To test the resistance, after withdrawal of sorafenib for 12 days, the *IC*_50_ of RENCA^R^ cells and RENCA^WT^ cells were re-determined, and the resistance index remained approximately 2-fold, confirming the stability of the resistant phenotype.

### 2.3. Cell Counting Kit-8 (CCK-8) Assay

Renal cell carcinoma (RCC) cells were seeded into 96-well plates at a density of 6000 cells per well and treated with the indicated drugs. A total of 110 μL of a 1:10 mixture of CCK-8 reagent (B34302, Bimake, Shanghai, China) and culture medium was added to each well. After incubation at 37 °C for 1.5 h, absorbance at 450 nm was measured using a microplate reader (BioTek Epoch, Windsor, VT, USA). For combination treatment assays, RCC cells were seeded at the same density and administered with the corresponding drugs. CCK-8 preparation and administration were performed as described above, and absorbance at 450 nm was measured every 24 h for 4–5 consecutive days. All cytotoxicity assays were performed at least in triplicate. The cytotoxicity of each compound was expressed as the half-maximal inhibitory concentration (*IC*_50_), defined as the drug concentration required to inhibit 50% of tumor cell growth. *IC*_50_ values were calculated using GraphPad Prism software (version 10.6.1, GraphPad Inc, MA, USA.).

### 2.4. Colony Formation Assay

RENCA^WT^ or RENCA^R^ cells were seeded into 6-well plates at a density of 500 cells per well and cultured in a complete medium or a complete medium containing sorafenib or 1D228. After incubation at 37 °C in a humidified incubator with 5% CO_2_ for 14 days, cells were washed with phosphate-buffered saline (PBS), fixed with 4% paraformaldehyde for 15 min, and stained with 0.1% crystal violet for 20 min. Plates were rinsed with distilled water and air-dried. Colonies were imaged and counted for quantitative analysis.

### 2.5. Wound-Healing Assay

RENCA^WT^ or RENCA^R^ cells were seeded into 6-well plates at a density of 1 × 10^5^ cells per well and cultured to 100% confluence. OSRC-2 or 786-O cells were seeded at 6 × 10^5^ cells/well in 6-well plates. A linear scratch was created using a 200 μL pipette tip. After washing with PBS, cells were cultured in 2% serum medium or 2% serum medium containing sorafenib and/or 1D228 to suppress proliferation. Images of the scratched areas were captured at 0 h, 24 h, and 48 h using an inverted microscope (Olympus CKX41, Tokyo, Japan). Wound closure rate was calculated as follows: wound closure = (initial scratch width − scratch width at 24 h or 48 h)/initial scratch width.

### 2.6. Cell Migration Assay

Cell migration assays were performed using 24-well Boyden chambers with 8 μm pore size (#353097, Corning Inc., New York, NY, USA). RCC cells were digested and resuspended in a complete medium at a density of 5 × 10^5^ cells/mL. A total of 500 μL of a complete medium or a medium containing sorafenib and/or 1D228 was added to the lower chamber, and 200 μL of cell suspension was added to the upper chamber. After incubation for 48 h, non-migrated cells on the upper surface were removed with cotton swabs. Migrated cells were fixed with methanol for 5 min and stained with 0.1% crystal violet for 20 min. Chambers were thoroughly rinsed with water and air-dried. Migrated cells were imaged and counted in randomly selected fields under an IX71 inverted microscope (Olympus, Tokyo, Japan).

### 2.7. Flow Cytometry

Cells were seeded into 6-well plates at 60% confluence and treated with the indicated drugs for 72 h. Both adherent and floating cells were collected for analysis. After trypsinization and resuspension, cells were collected and processed according to the manufacturer’s instructions for the Annexin V-FITC/PI apoptosis detection kit (MA0220-2, Meilunbio, Dalian, China). Briefly, cells were resuspended in binding buffer and stained with Annexin V-FITC and propidium iodide (PI). Following incubation in the dark at room temperature, apoptosis was analyzed using a flow cytometer (Beckman Coulter, Brea, CA, USA).

### 2.8. Animal Experiments

All mouse experiments were approved by the Binzhou Medical University Animal Ethical Committee and performed in accordance with institutional guidelines including the 3R principles. Female wild-type BALB/c mice (4–5 weeks old) were used and purchased from Shanghai Southern Model Biotechnology Co., Ltd. (Shanghai, China). All mice were housed in an SPF barrier system under constant environmental conditions: temperature 22–25 °C, humidity 50 ± 10%, and a 12h/12h light/dark cycle, with free access to food and water. After three days of acclimatization to eliminate stress-related interference, the experiments were formally initiated. In general, 8–10 female BALB/c mice were randomly allocated to each experiment group. The humane end point was followed by NIH Guidelines for Humane Endpoints. After tumor inoculation, healthy animals with robust tumors were randomized into each group for subsequent experiment. Exclusion criteria were defined as follows: (1) those mice that did not develop tumors were excluded from the experiment; (2) mice that died during the experimental period; and (3) mice that were euthanized prematurely due to reaching humane endpoints (e.g., sick animals or animals bearing over-sized tumours). For any mouse meeting the exclusion criteria, all corresponding data points recorded throughout the study (including body weight, tumor volume measurements, and blood biochemical parameters) were completely excluded from the final statistical analysis. Sample size was determined by referring to high-quality peer-reviewed studies in the field and further validated using the resource equation method. This approach ensured statistical validity of the experimental data and complied with the reduction principle of animal ethics. The total number of mice used in this study was 110.

For the establishment of subcutaneous drug-resistant tumor models, RENCA^WT^ cells were harvested at 70–80% confluence, centrifuged at 1000 rpm/min for 4 min, resuspended in sterile PBS at a density of 1 × 10^6^ cells per 100 μL, and kept on ice to maintain viability. The dorsal hair of the mice was shaved before the experiment, and 100 μL of cell suspension was subcutaneously inoculated into the right flank under anesthesia using isoflurane at a concentration of 3.5% with 400 mL air flow per minute. When tumor volume reached approximately 150 mm^3^, mice were administered with 60 mg/kg sorafenib (dissolved in autoclaved water) daily by oral gavage for 5 days, followed with a 3-day drug-free interval to avoid long-term treatment-induced toxicity. This on-off drug schedule was repeated for a total of 5 cycles. During the induction period, tumors were dissected and reinoculated into new BALB/c mice, when the parental tumors reached 1000 mm^3^. The treatment and tumor re-inoculation were repeated for 40 days (5 on–off drug treatments). Then, the tumors were harvested and reinoculated for testing drug resistance, and resistant tumor tissues were re-inoculated by tumor tissue transplantation to establish resistant tumor models for subsequent animal experiments.

Subcutaneous tumor tissue transplantation: Recipient BALB/c mice were acclimatized before transplantation. The mice were anesthetized using isoflurane, and the skin on the lateral thigh root was gently pinched, and a 0.5 cm longitudinal micro-incision was made using sterile ophthalmic scissors. A complete subcutaneous pocket (0.5–1.0 cm deep) was created by blunt dissection along the superficial fascia, avoiding muscle layers and blood vessels throughout the procedure. A sterile piece of tumor tissue (1–2 mm^3^) was carefully implanted into the subcutaneous pocket using sterile forceps, keeping the tumor graft closely attaching the subcutaneous tissue without dislodgement. The surgical wound was disinfected with iodine and allowed to heal naturally. Wound healing was observed daily post-transplantation. The formation of a palpable subcutaneous hard nodule within 5–7 days was considered to be a successful engraftment, and the next round of drug selection was then initiated. After completing five cycles of drug administration, the stable drug-resistant tumor was established. Tumor tissues were tested and named RENCA^RT^ (sorafenib-resistant). The resistant tumor tissues were re-inoculated into new mice to maintain the tumors and for subsequent experiments. Resistance criteria: Successful establishment of an acquired resistance model was defined by the absence of significant growth inhibition of RENCA^RT^ tumors following sorafenib treatment or significantly decreased inhibition rate compared with the wild-type tumors treated with sorafenib.

Drug treatment: Healthy mice or those with subcutaneous tumor volumes that reached 150–200 mm^3^; the mice were randomly assigned to groups. Mice in each group were administered via oral gavage with sterile water (vehicle), 60 mg/kg sorafenib, or 1D228 at different doses that were indicated in figure legends, with a uniform dosing volume of 100 μL. All drugs were administered via oral gavage at the same fixed time daily. During the experiment, tumor length and width were measured every two days, and tumor volume was calculated using the formula V = 0.5 × length × width × width. Mice presenting with wound infections, tumor ulcerations, severe weight loss, or a moribund state were promptly removed from the experiment. All experimental data were recorded throughout the study. At the experimental endpoint, mice were euthanized by cervical dislocation, and intact tumor tissues were excised, photographed, and weighed. Collected tumor tissues were used for immunohistochemistry and Western blot analyses.

### 2.9. Immunohistochemistry

Tissue sections (5 μm thick) were heated in a 65 °C oven for 2 h. Sections were deparaffinized in xylene and rehydrated through a graded ethanol series (95%, 85%, 75%). Antigen retrieval was performed by microwave heating in a sodium citrate buffer (C1010, Solarbio), followed by natural cooling down to room temperature. Endogenous peroxidase activity was blocked with 3% hydrogen peroxide at room temperature for 15 min. Sections were permeabilized with 0.2% Triton X-100 (MB2486, Meilun Bio, Dalian, China) at room temperature for 10 min and blocked with 10% fetal bovine serum or goat serum at 37 °C for 45 min. Sections were incubated with primary antibodies diluted in blocking buffer overnight at 4 °C. After washing three times with PBS, sections were incubated with corresponding secondary antibodies at 37 °C for 1 h. After PBS washing, sections were stained with DAB for 1–2 min, counterstained with hematoxylin, dehydrated, and mounted with resin. Antibodies and kits used: DAB kit (GK60051, Shanghai, China); CC3 (CST 9661T, 1:200, Danvers, MA, USA); and Ki67 (CST 9129S, 1:200, Danvers, MA, USA). Microscopic images were acquired for further analysis.

### 2.10. Western Blotting

RCC cells were washed with PBS and digested with 0.25% trypsin in PBS (T8150, Solarbio, Beijing, China) for 1 min. Cell pellets were collected by centrifugation at 1000 rpm for 4 min. Protein samples were extracted using lysis buffer (P0013, Beyotime, Shanghai, China) supplemented with protease inhibitor cocktail (B15001, 100:1 dilution, Bimake, Shanghai, China) and phosphatase inhibitor cocktail (HY-K0022, 100:1 dilution, MCE, Shanghai, China). Lysates were centrifuged at 12,000× *g* for 20 min at 4 °C, and protein concentration was determined using a BCA protein assay kit (P0012, Beyotime, Shanghai, China). Protein samples were denatured by boiling at 95 °C for 10 min in loading buffer. Equal amounts of protein (20–50 μg per lane) were separated on 8–12.5% gels which were prepared using Omni-Easy™One-Step PAGE Gel Fast Preparation Kit (SL1130, Coolaber, Beijing, China or PG212, Epizyme, Shanghai, China). After PAGE, the proteins were transferred to PVDF membranes (ISEQ00010/IPVH00010, Immobilon) using a standard wet transfer method in 20% methanol tris-glycine buffer at constant current at 200 mA, and 1 min per kDa. Membranes were blocked with 5% non-fat milk at room temperature for 2 h and incubated overnight at 4 °C with primary antibodies against: p-c-Met (3077S, Immunoway, Newark, DE, USA), c-Met (25869-1-AP, Proteintech, Rosemont, IL, USA), p-AXL (AF8523, Abmart, Shanghai, China), AXL (AF7793, Affinity, Cincinnati, OH, USA), AXL (8661S, CST, Danvers, MA, USA), p-AKT (Ser473, T40067F, Abmart, Shanghai, China), p-AKT (Thr305/308/309,PC5182, Abmart, Shanghai, China), AKT (T55b1F, Abmart, Shanghai, China), p-PI3K (YP0765, Immunoway, Newark, DE, USA), PI3K (4292S, CST, Danvers, MA, USA), p-4EBP-1 (2855T, CST, Danvers, MA, USA), 4EBP-1 (60246-1-Ig, Proteintech, Rosemont, IL, USA), p-70S6K (9234T, CST, Danvers, MA, USA), and 70S6K (9202S, CST, Danvers, MA, USA). All aforementioned antibodies are polyclonal antibodies and were diluted 1:1000 to 1:2000. Membranes were washed three times with TBST and incubated with horseradish peroxidase (HRP)-conjugated secondary antibodies (goat anti-rabbit IgG (H&L), HRP, 1:5000, GenScript; goat anti-mouse IgG (H&L), HRP, 1:5000, GenScript, Piscataway, NJ, USA) at room temperature. Protein bands were visualized using an enhanced chemiluminescence (ECL) reagent (YEASEN, 36208ES60, Shanghai, China). For protein extraction from mouse tumor tissues, 35 mg of tumor tissue from each group was minced and lysed in RIPA lysis buffer (MA0151, Meilun Bio, Dalian, China) supplemented with protease inhibitor cocktail (MA0001, 100:1 dilution, Meilun Bio, Dalian, Cina) and phosphatase inhibitor cocktail (HY-K0022, 100:1 dilution, MCE, Shanhai, China).

### 2.11. RNA Sequencing (RNA-Seq) and Data Analysis

Cell samples from RENCA^WT^ and RENCA^R^ were collected at the mid-logarithmic phase and subjected to further processing, with three samples collected per group and sent to the company for subsequent process. Tumor samples were collected from sensitive or resistant RENCA tumor tissues. Total RNA was extracted from tissues using TRIzol reagent. Approximately 35 mg of tumor tissue samples were excised, fragmented, and subjected to homogenization using a glass homogenizer in TRIzol reagent to isolate total RNA from tumor tissues. RNA-seq analysis was performed by Beijing Boyun Huakang Gene Technology Co., Ltd. (Beijing, China). Briefly, after RNA extraction, purification and library construction, paired-end (PE) sequencing was performed on these libraries using high-throughput sequencing platforms based on DNBSEQ or Illumina, followed by filtering of the raw sequencing data. Two main normalization algorithms were employed: the primary quantitative metric was FPKM, which corrects for the confounding factors of sequencing depth and gene length; raw read counts were retained as auxiliary data for subsequent differential expression analysis. The high-quality clean reads obtained were aligned to the human/mouse reference genome. Gene expression levels were quantified based on the alignment results. Subsequently, differential expression analysis, enrichment analysis and clustering analysis of the samples were further performed. Differential gene detection was performed using four algorithms: DESeq2, EBSeq, NOIseq, and PoissonDis, each with fixed filtering thresholds (fold change + statistical test). The raw sequencing data generated in this study have been deposited in the NCBI Sequence Read Archive (SRA) under BioProject accession number SRP717053. The dataset comprises 14 samples in total, including 6 mouse eukaryotic cell samples and 8 mouse tumor tissue samples, all in *.fastq.gz format.

### 2.12. Whole-Mount Immunofluorescence

On day 1, tumor tissues were fixed in 4% paraformaldehyde at 4 °C overnight. On day 2, tissues were sectioned into thin slices, washed with PBS for 30 min, digested with 20 μg/mL proteinase K for 5 min, and permeabilized with 100% methanol at room temperature for 30 min. Tissue sections were washed with PBS for 30 min at room temperature and blocked overnight at 4 °C in PBS containing 3% non-fat milk and 0.3% Triton X-100. On day 3, sections were incubated with primary antibody against CD31 (RD-AF3628, R&D Systems, Minneapolis, MN, USA) diluted 1:400 at 4 °C overnight (≥16 h). On day 4, tissues were washed with PBS containing 0.3% Triton X-100 at 4 °C for 1.5 h and blocked in PBS containing 3% non-fat milk and 0.3% Triton X-100 at room temperature for 1.5 h. Sections were incubated with secondary antibody (goat anti-rabbit IgG, AbbKine-A23330, Wuhan, China) diluted 1:200 at room temperature for 2 h. Tissues were washed with PBS containing 1.5% non-fat milk and 0.3% Triton X-100 for 1 h at room temperature, then washed overnight at 4 °C in PBS containing 0.3% Triton X-100. Samples were mounted using Vectashield mounting medium (H-1000, Vector Laboratories, Burlingame, CA, USA). Fluorescence signals were detected using a confocal laser scanning microscope (Zeiss LSM880 with Airyscan, Oberkochen, Germany).

For the data acquisition and analysis process, we cut 2–3 pieces of thin tissue from each tumor and mixed the same group’s tumor pieces into one tube for staining. Thus, a total of 12–18 pieces from 6 tumors were prepared for each treatment group. During image collection, one field of each tumor piece was imaged. Finally, 10–18 images were collected for each group (some tumor tissues failed the staining or it was impossible for them to undergo imaging due to the destruction of tissue morphology). These images were imported into Adobe Photoshop, and the red pixel value was recorded for each field. The total red pixel value was then converted to area by multiplying the pixel to area conversion factor (actual area/total pixels). The final area values were then imported into GraphPad Prism for statistical analysis. The analysis was not performed in a blind fashion but analyzed by two different students.

### 2.13. Statistical Analysis

Statistical analyses were performed using GraphPad Prism 10.6.1 software. Continuous variables were expressed as mean ± standard deviation (SD). Comparisons between two groups were analyzed using Student’s t-test, and multiple comparisons were performed using one-way analysis of variance (one-way ANOVA). Statistical significance was defined as follows: ns, not significant, *, *p* < 0.05; **, *p* < 0.01; ***, and *p* < 0.001.

### 2.14. Hematoxylin and Eosin (H&E) Staining

Mice were euthanized by cervical dislocation, and the thoracic cavity was opened. A needle was inserted approximately 3 mm into the left ventricle, and cardiac perfusion was performed sequentially with phosphate-buffered saline (PBS) and 4% paraformaldehyde. Perfusion was terminated when the lungs turned pale and the liver showed discoloration. The heart, liver, spleen, lungs, and kidneys were then collected, fixed in 4% paraformaldehyde overnight at 4°, dehydrated, embedded, and sectioned. Tissue sections of 5 μm thickness were prepared and baked at 75 °C for 1 h, followed by deparaffinization and graded rehydration according to previously established protocols. The sections were stained with hematoxylin for 5 min, washed for 10 min, differentiated in acid-alcohol for 3 s (Cat. No. C0163M, Beyotime Biotechnology, Shanghai, China) and washed for 10 min. Counterstaining was performed with eosin for 3 min, followed by another 10 min rinse under running water. The sections were then dehydrated through a graded ethanol series, cleared, and mounted with resin. Histological observations and analyses were performed using a Zeiss microscope (Axio Observer 5, Oberkochen, Germany) equipped with a color camera (Axiocam 208, Oberkochen, Germany) and a monochrome camera (Axiocam 705, Oberkochen, Germany).

### 2.15. Blood Chemistry Tests

Blood samples were collected through orbital veins after sacrificing mice with overdose of isoflurane. The 5 mL coagulation-promoting tubes (Huabo Medical Devices (Shandong) Co., Ltd., Yantai, China; Registration No.: Lu 20192220597) and some 1.5 mL anticoagulant tubes (RZ-20211215-C2, Supin, Nantong, China) were prepared for serum or blood collection. Approximately 100 μL of blood was first collected into the anticoagulant tube, and the remaining 300–500 μL was collected into the coagulation-promoting tube. The coagulation-promoting tube was gently inverted 3–4 times to ensure thorough mixing of the blood with the clotting activator (RT). Whole blood samples were analyzed within 1 h using an automated hematology analyzer (HEMAVET 950FS, Drew Scientific, Waterbury, CT, USA) for complete blood count (CBC) parameters. Serum samples were allowed to stand at room temperature for 30 min and then centrifuged at 3000 rpm for 20 min at room temperature. The resulting serum was transferred to a new 1.5 mL microcentrifuge tube and kept on ice. For serum samples with insufficient volume, PBS dilution was performed and the dilution factor was recorded for subsequent calculations. Serum biochemical parameters were measured using an automated biochemical analyzer (CS-600B, DIRUI Industrial Co., Ltd., Changchun, China), and this required a minimum serum volume of 200 μL. The hematological and serum biochemical data were then imported into GraphPad Prism (version 10.6.1, GraphPad Inc, MA, USA.) for statistical analysis.

## 3. Results

### 3.1. Induction of Sorafenib-Resistant RCC Cell Model

**Figure 1:**
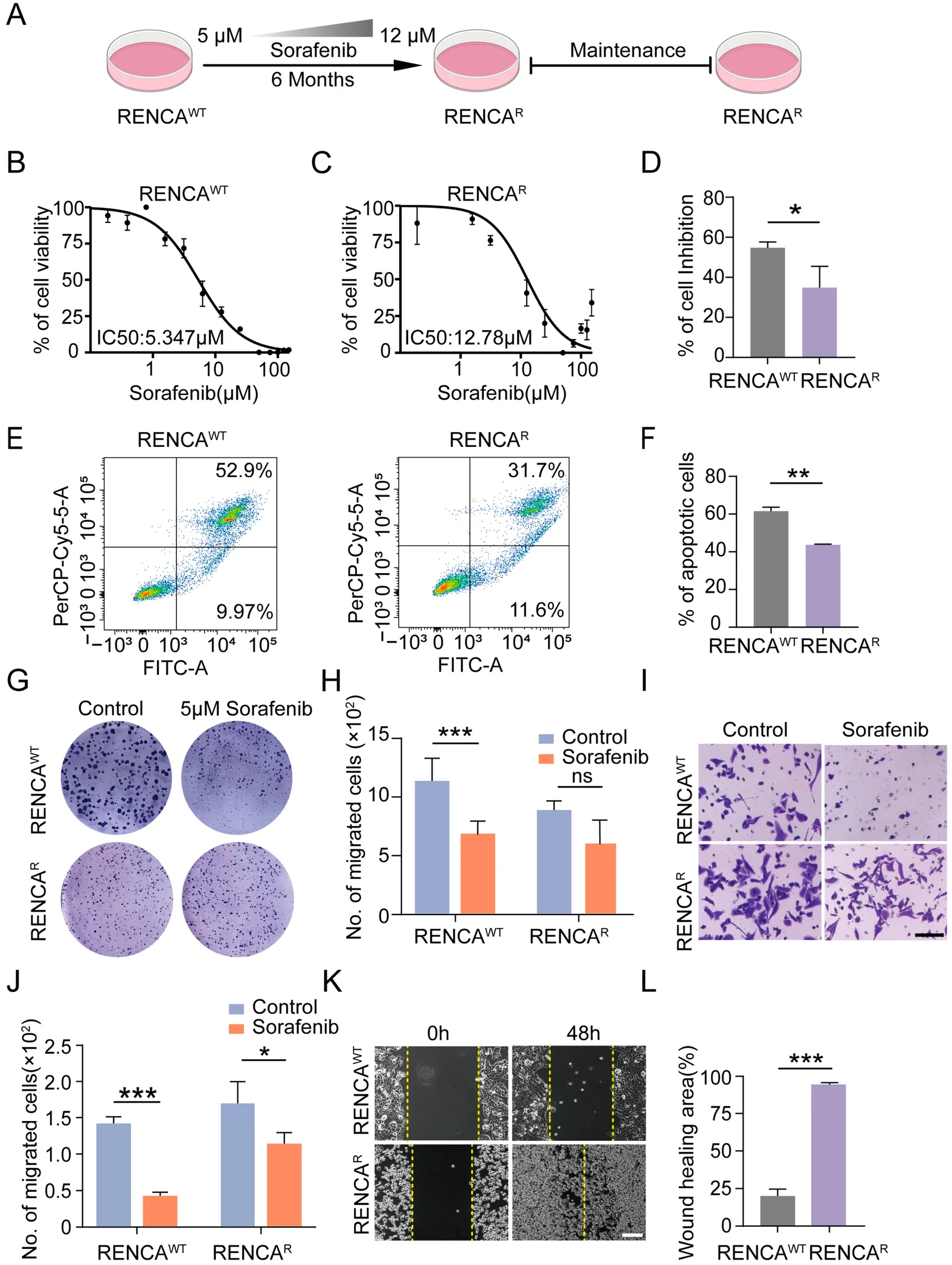
Establishment of sorafenib-resistant renal cell carcinoma cell model. (**A**) Wild-type RENCA cells (RENCA^WT^) were continuously cultured with sorafenib at concentrations ranging from 5 μM to 12 μM using a concentration gradient escalation method over a period of 6 months to establish a sorafenib-resistant RENCA cell line (RENCA^R^). Subsequently, the resistant cell line was maintained in a culture medium containing sorafenib to preserve the resistant phenotype, thereby obtaining a stable drug-resistant cell line. (**B**) Determination of the *IC*_50_ in RENCA^WT^ cells using the CCK-8 assay. *n* = 3. The concentrations of sorafenib were 0, 0.09, 0.19, 0.39, 0.78, 1.56, 3.125, 6.25, 12.5, 25, 50, 75, 100, 125, and 150. (**C**) Assessment of sorafenib resistance in RENCA^R^ cells by *IC*_50_ by CCK-8 assay, *n* = 3. (**D**) Quantification of cell inhibition rate of RENCA^WT^ and RENCA^R^ cells treated with 5 μM sorafenib, *n* = 3 per group. (**E**,**F**) Quantitative analysis of apoptotic cell percentages in RENCA^WT^ and RENCA^R^ cells treated with 5 μM sorafenib detected by Annexin V/PI double staining, *n* = 3 per group. (**G**,**H**) Colony assay of RENCA^WT^ and RENCA^R^ cells treated with 5μM sorafenib, *n* = 3 per group. (**I**,**J**) Detection of cell mobility of RENCA^WT^ and RENCA^R^ cells under the treatment of 5 μM sorafenib by transwell assay, *n* = 3 per group. Bar: 50 mm. (**K**,**L**) Representative images (**K**) and quantitative wound healing rates. Yellow dotted lines indicate the scratch boundary. (**L**) of the scratch assay assessing the inhibition of sorafenib on cell migration of RENCA^WT^ and RENCA^R^ cells, *n* = 3 per group. Bar: 50 μm. Data in (**D**,**F**,**L**) were analyzed using Student’s t-test. Data in (**H**,**J**) were analyzed using two-way ANOVA. All data are presented as mean ± SD. ns, not significant, *, *p* < 0.05; **, *p* < 0.01; ***, and *p* < 0.001. Groups: Control (RENCA^WT^), RENCA^R^ (**D**–**F**). RENCA^WT^-Control, RENCA^WT^-sorafenib, RENCA^R^-Control, and RENCA^R^-sorafenib (**G**–**L**).

To study the mechanisms and strategies to overcome drug resistance in RCC, we developed a sorafenib-resistant murine RCC cell line RENCA^R^. The RENCA cells were exposed to progressively elevated sorafenib (5–12 μM) over the treatment time-course. (Figure 1A). After 6 months’ induction, RENCA cells developed sorafenib resistance and were maintained in a culture medium containing sorafenib. Cell viability assay by CCK-8 showed that the half-maximal inhibitory concentration (*IC*_50_) of sorafenib on resistant RENCA cells (RENCA^R^) was at 12.78 μM, which was more than twice as much as that on wild-type RENCA cells (RENCA^WT^) at 5.35 μM (Figure 1B,C). Quantification of the inhibition rate of sorafenib demonstrated a significant decrease in RENCA^R^ cells compared to RENCA^WT^ cells (Figure 1D). To keep the resistant phenotype of RENCA^R^ cells, we routinely maintained the resistant cells in the 7 μM sorafenib culture medium. To test whether the resistance remained stable, we did *IC*_50_ again on the 12th day after sorafenib withdrawal in RENCA^R^ cells. Notably, the *IC*_50_ value of RENCA^R^ cells (13.31 μM) remained twice as much as that of wildtype cells (5.70 μM), which is the same as before withdrawal (Figure S1A,B). Further analysis of the cell death of RENCA cells under the treatment of sorafenib by flow cytometry found a sharp drop in total apoptotic cell population from 62.87% in RENCA^WT^ cells to 43.3% in RENCA^R^ cells, suggesting a refractory phenotype to sorafenib treatment (Figure 1E,F). In the colony assay, sorafenib exhibited strong inhibition on colony formation on RENCA^WT^ cells, whereas the RENCA^R^ cells did not respond to sorafenib treatment, suggesting resistance to sorafenib (Figure 1G,H). In addition to enhanced cell proliferation of RENCA^R^ cells, the cell migration assay by transwell experiments demonstrated the maintenance of cell mobility in RENCA^R^ cells under the treatment of sorafenib where the migration of RENCA^WT^ cells was impaired significantly (Figure 1I,J). Similarly, in the scratch assay, RENCA^R^ cells recovered the gap completely in 48 h, when the scratch gap in RENCA^WT^ cells remained largely open (Figure 1K,L). These data demonstrated that we have successfully established the sorafenib-resistant RCC cell line, which will be applied to further experiments for mechanistic investigations.

### 3.2. The Combination Treatment of Compound 1D228 and Sorafenib-Inhibited Sorafenib-Resistant RENCA^R^ Cell Proliferation and Migration

**Figure 2:**
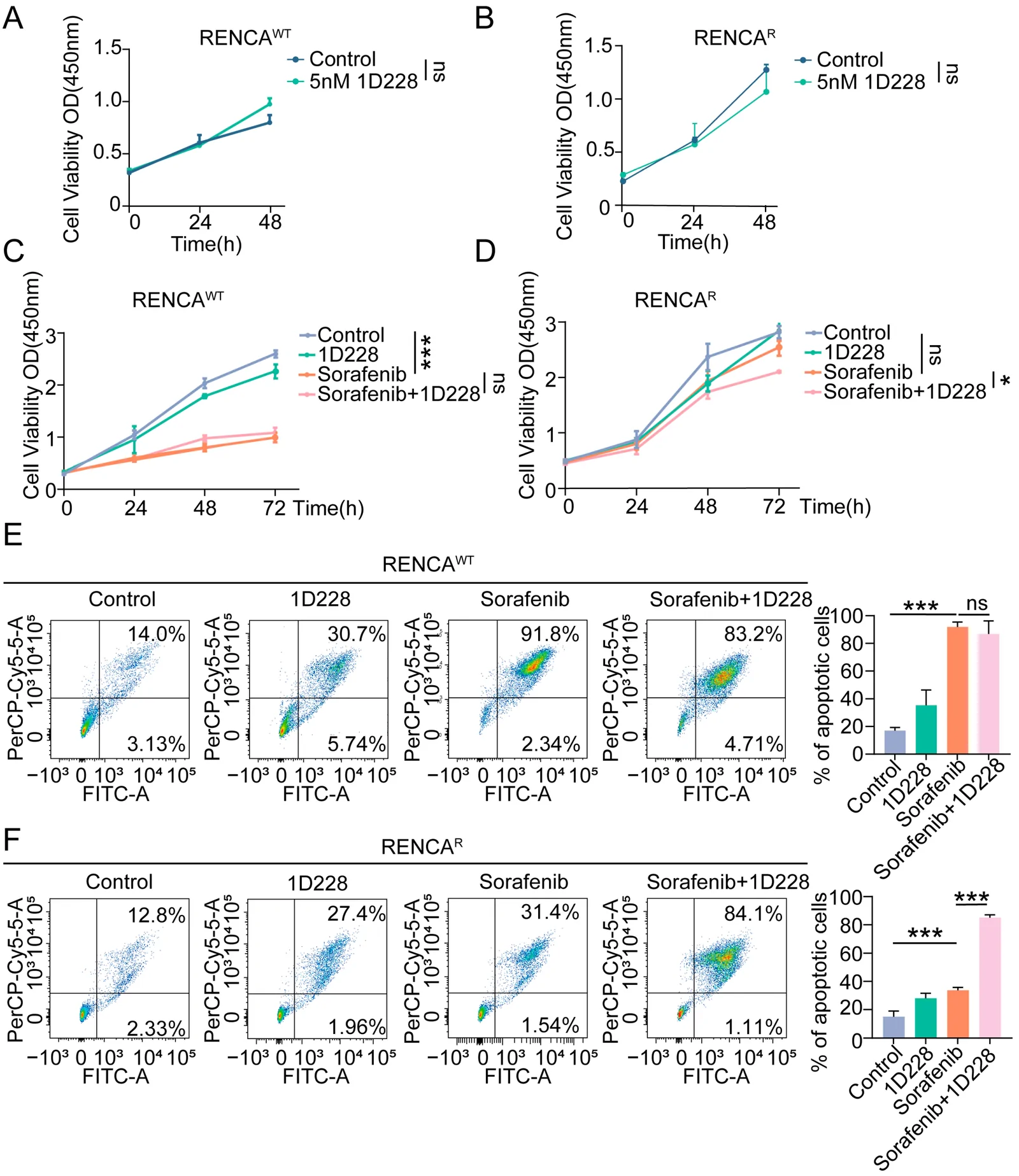
The combination of 1D228 and sorafenib suppressed RENCA^R^ cell proliferation and induced apoptosis. (**A**,**B**) Cell proliferation by CCK-8 assay in RENCAWT (**A**) and RENCA^R^ (**B**) cells following treatment with different concentrations of 1D228, *n* = 3 per group. (**C**,**D**) Proliferation of RENCA^WT^ (**C**) and RENCA^R^ (**D**) cells treated with control, 1D228, sorafenib, or 1D228 plus sorafenib, *n* = 3 per group. Sorafenib: 5 μM. 1D228: 5 nM. (**E**,**F**) Apoptosis analysis of RENCA^WT^ (**E**) and RENCA^R^ (**F**) cells in each group by flow cytometry. The percentage of apoptotic cells was quantified. RENCAWT: *n* = 6, RENCAR: *n* = 3. (**A**–**D**) were analyzed using two-way ANOVA. Data in (**E**,**F**) were analyzed using one-way ANOVA. All data are presented as mean ± SD. ns, not significant; *, *p* < 0.05; ***, and *p* < 0.001. Groups: control (DMSO); 1D228 (**A**,**B**). Control, sorafenib, 1D228, and combination (**C**–**F**).

In our previous study, we developed a tepotinib-derived compound 1D228, which showed great inhibition on hepatocellular carcinoma (HCC) and gastric cancer cells by selectively targeting kinases c-Met and AXL. More importantly, 1D228 is more potent than Tepotinib and did not show any acute toxicity at low dose [49]. Based on these findings, we investigated whether 1D228 also has an inhibitory effect on sorafenib-resistant RCC where c-Met and AXL might be activated. According to our previous experience in HCC, the *IC*_50_ of c-Met inhibition by 1D228 was 0.98 nM. Thus, we tested 1D228 in RENCA cells with a series of concentrations around 0.98 nM. We chose the concentration of 0.25 to 5 nM to treat both RENCA^WT^ and RENCA^R^ cells. Interestingly, the wild-type and sorafenib-resistant cells did not respond to 1D228 significantly, and the cell viability remained the same as the control treatment (Figure S1, Figure 2A,B). These results suggest that these concentrations of 1D228 did not have an impact on RCC proliferation. Further investigation with combination treatment of sorafenib at 5 μM and 1D228 at 5 nM showed that RENCA^WT^ cells were not further inhibited compared with sorafenib treatment alone**,** suggesting that 1D228 has no effect on wild-type cells (Figure 2C). However, the RENCA^R^ cells showed a significant decrease in cell proliferation under the combination treatment compared with sorafenib treatment alone (Figure 2D). Furthermore, when we tested cell apoptosis by flow cytometry using PI and Annexin V staining, there was a sorafenib-induced dramatic increase in the total apoptotic cell population in RENCA^WT^ cells. But the combination treatment produced only a slight increase, which did not reach statistical significance (Figure 2E). On the contrary, RENCA^R^ did not respond to sorafenib as much as RENCA^WT^ cells did, and only 32.91% of the apoptotic cell population was observed in resistant cells vs. 94.14% in wild-type cells. Interestingly, the combination treatment of sorafenib and 1D228 greatly increased the apoptosis to a similar level as in RENCA^WT^ cells that were treated with sorafenib alone or sorafenib combined with 1D228 (Figure 2F). Notably, 1D228 increased apoptosis in both RENCA^WT^ and RENCA^R^ cells, which might be due to other potential kinase targets being inhibited by 1D228. These results indicated that 1D228 could attenuate RENCA^R^ cells to sorafenib treatment.

**Figure 3:**
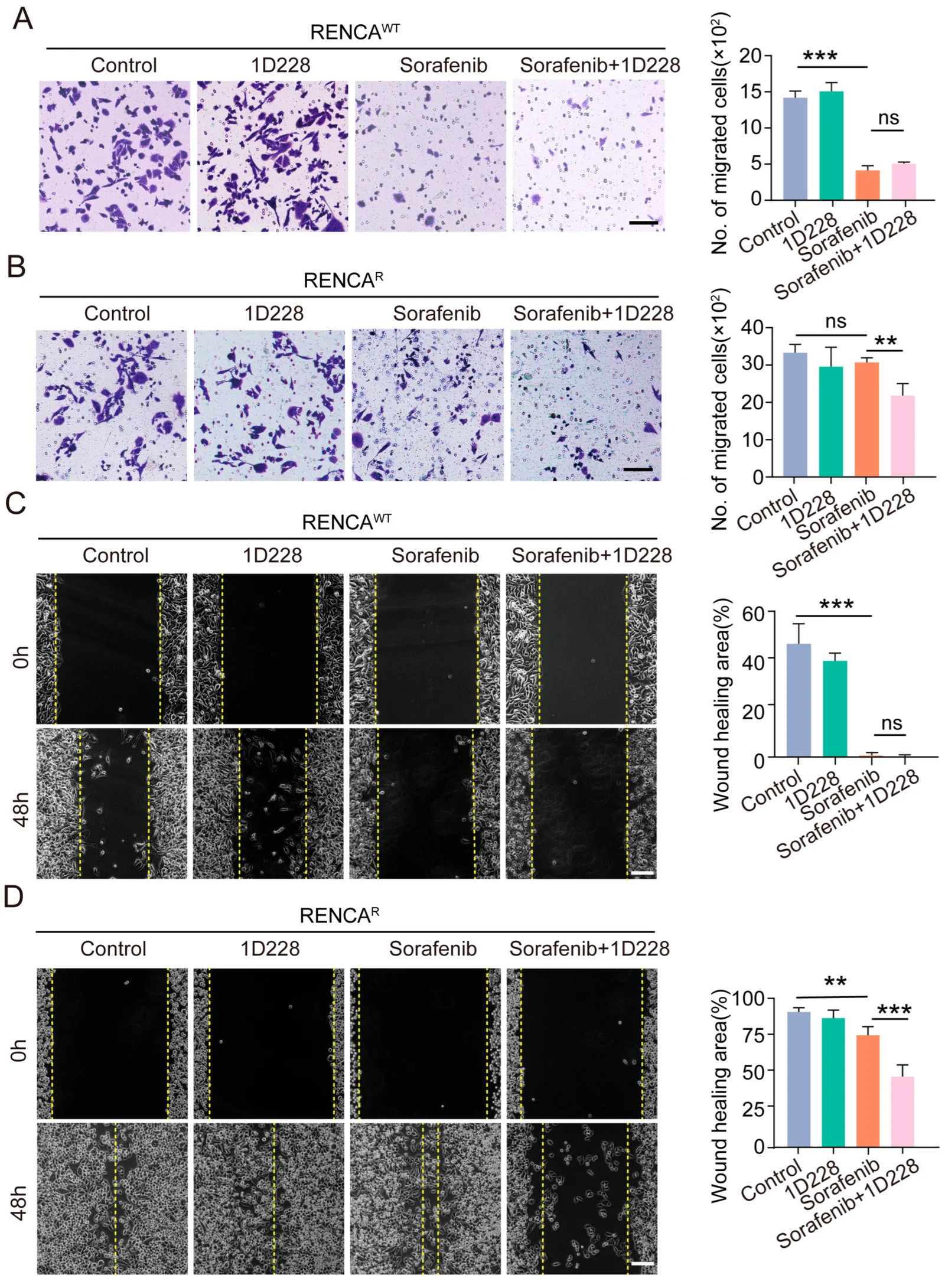
The combination of 1D228 and sorafenib suppressed RENCA^R^ cell migration. (**A**,**B**) Transwell assays were performed to evaluate RENCA^WT^ (**A**) and RENCA^R^ (**B**) cell migration. Representative images showed migrated RCC cells after treatment. Images from each group were used to quantify the numbers of migrated and invaded cells, *n* = 3. Bar: 50 μm. (**C**,**D**) Scratch assay of RENCA^WT^ (**C**) and RENCA^R^ (**D**) cell migration in different groups. Yellow dotted lines indicate the scratch boundary. The percentage of wound closure was quantified for each group, *n* = 3. All data were analyzed using one-way ANOVA and presented as mean ± SD. Bar: 50 mm. ns, not significant, **, *p* < 0.01; ***, and *p* < 0.001. Groups: control, 1D228, and sorafenib or 1D228 plus sorafenib.

Cancer cell mobility is essential for tumor metastasis. Therefore, we examined the migration of RENCA cells. In the transwell assay, we found that sorafenib significantly inhibited cell migration, but 1D228 did not produce further inhibition in RENCA^WT^ cells (Figure 3A). However, in the RENCA^R^ cells, 1D228 combined with sorafenib significantly blocked cell migration (Figure 3B). Similarly, the scratch assay also demonstrated that the combination of 1D228 and sorafenib could produce enhanced inhibition on cell mobility in RENCA^R^ cells, but not in RENCA^WT^ cells (Figure 3C,D). Again, these data suggested that 1D228 may enhance the response of resistant RCC cells to sorafenib treatment.

### 3.3. D228 Partially Overcome the Tumor Resistance in Sorafenib Refractory RCC Tumor Models

**Figure 4:**
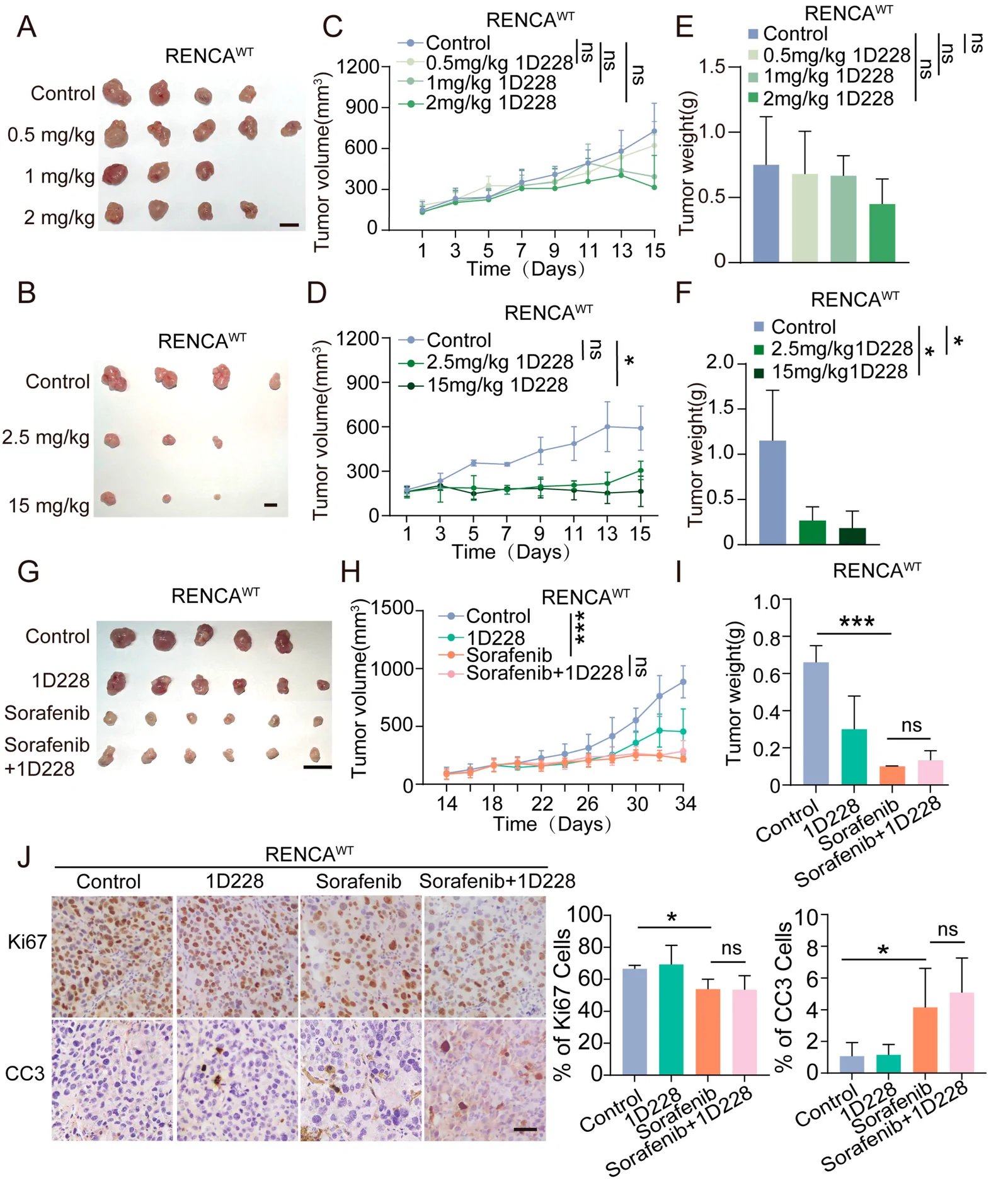
1D228 suppresses RENCA^WT^ tumor growth in a dose-dependent manner. (**A**–**F**) RENCA xenograft tumor images, tumor growth curve, and tumor weight under 1D228 treatment. Groups: Control, 0.5 mg/kg 1D228, 1 mg/kg 1D228, 2 mg/kg 1D228, *n* = 3–5. Groups: Control, 2.5 mg/kg 1D228, 15 mg/kg 1D228, *n* = 3–4. The total number of mice in the study was 26. Bar: 5 mm. (**G**–**I**) Representative tumor images (**G**), tumor growth curves (**H**), and tumor weight (**I**) from each group treated with control (autoclaved distilled water), 1D228 (2 mg/kg), sorafenib (60 mg/kg), or 1D228 plus sorafenib. The total number of mice in the study was 23. *n* = 5–6 per group. Bar: 5 mm. (**J**) Immunohistochemistry (IHC) staining was performed to detect Ki67 and CC3 positive cells in RENCAWT tumor tissues. Positive cell percentage was quantified for each group. *n* = 8 (Ki67), *n* = 5–6 (CC3) images per group. Bar: 50 μm. All data were analyzed using one-way ANOVA. All data are presented as mean ± SD. ns, not significant; *, *p* < 0.05; ***, and *p* < 0.001.

In our previous studies, 1D228 was used to treat different tumors at doses from 2 mg/kg to 8 mg/kg and demonstrated significant inhibition on tumor growth [49]. According to this experience, we treated wild-type tumors with 1D228 at doses ranging from 0.5 mg/kg to 15 mg/kg to investigate the impact of 1D228 on RCC tumors. Notably, wild-type RENCA tumors demonstrated a dose-dependent inhibition under the treatment of 1D228 (Figure 4A,B). Low doses from 0.5 mg/kg to 2 mg/kg did not show significant suppression on tumor growth and tumor weight, while the doses from 2.5 mg/kg to 15 mg/kg showed significant inhibition on tumor growth and tumor weight (Figure 4C–F). These data indicate that 1D228 could only produce inhibition on RCC tumors at high doses. Additionally, we checked again the toxicity of 1D228 by examining the organs by H&E staining. The results were consistent with our previous studies on HCC models that 1D228 did not alter primary organ morphologies in the heart, liver, spleen, lung, and kidney (Figure S2), indicating 1D228 may not induce acute organ toxicity. As 2 mg/kg 1D228 did not show a significant tumor inhibition effect, it is an ideal dose to study its effect in the combination treatment because we can rule out the direct impact by 1D228-induced inhibition. We treated the wild-type tumors with a combination of 1D228 and sorafenib. As expected, RENCA^WT^ tumors were super sensitive to sorafenib treatment, but did not respond to a low dose of 1D228 (2 mg/kg) treatment, and the combination treatment did not show further inhibition compared with sorafenib alone (Figure 4G–I). Consistent with the tumor growth and weight data, immunohistochemistry staining of the tumor proliferation marker Ki67 showed no differences between sorafenib and combination therapy. Cleaved caspase-3 staining also revealed that tumor apoptosis was not altered between sorafenib and combination therapy (Figure 4J). Again, 1D228 had no impact on RENCA^WT^ tumors. These data suggest that a low dose of 1D228 could not have a significant impact on sorafenib therapy, which is similar with the results obtained in the cell models.

**Figure 5:**
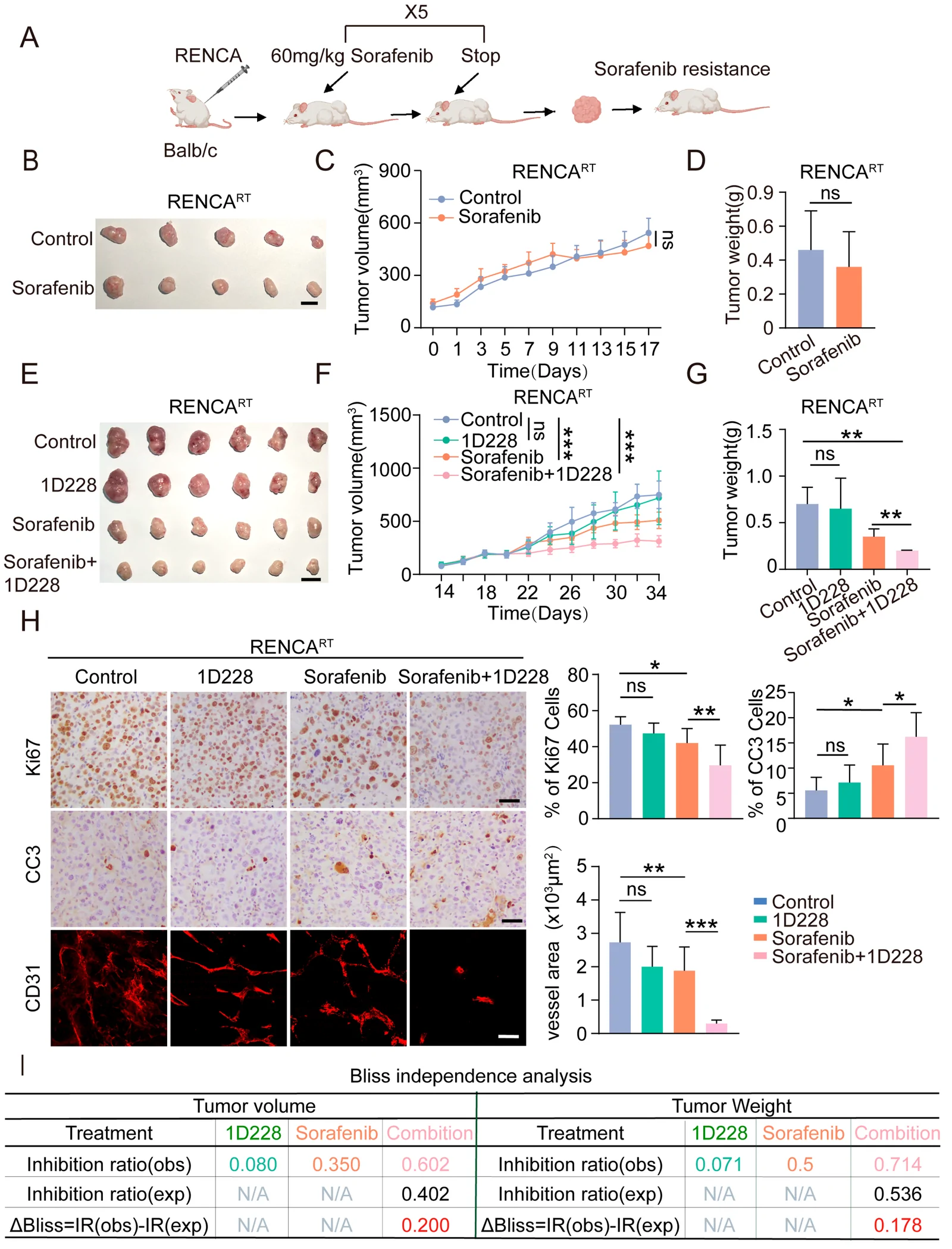
1D228 restores sorafenib sensitivity in resistant renal cell carcinoma. (**A**) Schematic diagram of the establishment procedure of a sorafenib-resistant RENCA^RT^ xenograft mouse model. RENCA^WT^ cells were subcutaneously implanted into BALB/c mice and intermittently treated with 60 mg/kg sorafenib. The resistant phenotype was acquired in vivo following repeated subcutaneous transplantation and drug stimulation (RENCA^RT^). (**B**–**D**) Representative images of sorafenib-treated RENCA^RT^ tumors (**B**), tumor growth (**C**), and tumor weight quantification at the experimental endpoint (**D**). Groups: Control, sorafenib, *n* = 5 mice per group. The total number of mice in the study was 10. Bar: 5 mm. (**E**–**G**) Representative images of RENCA^RT^ tumors from four treatment groups (control, 1D228, sorafenib, and sorafenib + 1D228) (**E**), tumor growth curves of each group (**F**), and tumor weight quantification at the endpoint (**G**). *n* = 6. The total number of mice in the study was 24. Bar: 5 mm. (**H**) Whole-mount and immunohistochemistry (IHC) staining was performed to detect Ki67, CC3, and CD31 in resistant tumor tissues from different treatment groups. Positive signals were quantified for each group. *n* = 10–13 (Ki67), *n* = 9 (CC3), *n* = 6 (whole mount) tumors per group. Bar: 50 μm. (**I**) Bliss independence analysis of the combination effect of 1D228 and sorafenib in RENCAR xenograft models. (**C**,**F**) data were analyzed using two-way ANOVA. Data in (**D**,**G**,**H**) were analyzed using one-way ANOVA. All data are presented as mean ± SD. ns, not significant; *, *p* < 0.05; **, *p* < 0.01; ***, and *p* < 0.001.

To study whether 1D228 could reproduce the suppression effect as we observed in RENCA^R^ cells, we developed a sorafenib-resistant tumor model by drug induction in vivo. As shown in Figure 5A, RENCA^WT^ cells were inoculated in BALB/c mice, and sorafenib at the dose of 60 mg/kg was administered intermittently (3 days on drug and 2 days off) when the tumors reached the size of around 200 mm^3^. After this drug administration for 5 cycles, the tumors developed the drug-resistant phenotype, which was verified by sorafenib treatment, showing no suppression on either tumor growth or tumor weight (Figure 5B–D). These results demonstrated the success of establishing the in vivo sorafenib-resistant RENCA tumor (RENCA^RT^).

Next, we administered 1D228 at a low dose to RENCA^RT^ tumor-bearing mice combined with sorafenib to test if 1D228 would overcome the sorafenib resistance. Interestingly, a low dose of 1D228 monotherapy did not show any inhibition on tumor growth; however, the combination therapy significantly reduced tumor growth and weight compared with sorafenib treatment alone (Figure 5E–G). Immunohistochemistry staining of the cell proliferation marker of Ki67 showed a large decrease in tumor cell proliferation under the treatment of sorafenib combined with 1D228, compared with sorafenib or 1D228 monotherapy. These data were consistent with the tumor growth results. Further analysis of cell apoptosis by cleaved caspase-3 staining demonstrated a significant increase in apoptosis in tumors receiving combination therapy (Figure 5H). Since sorafenib targets tumor angiogenesis, we examined tumor vessel density by whole-mount staining of CD31. Similarly, combination therapy almost vanished tumor vessels in RENCA^RT^ tumors, but 1D228 and sorafenib treatment did not change the vessels’ density markedly (Figure 5H). When we verified the synergism by combination treatment by bliss independence, the ΔBliss was 0.200 and 0.178 by tumor size or tumor weight respectively, which indicated a synergistic effect by combination treatment (Figure 5I). Taken together, these data suggest that the low dose of 1D228 produced synergistic effects with sorafenib in the suppression of resistant RCC tumor growth and angiogenesis, indicating that 1D228 could enhance the response of resistant RCC to sorafenib treatment. Notably, sorafenib treatment showed a decrease in the Ki67 positive cell population and an increase in cleaved caspase-3, which corresponded to the retarded tumor growth. This might be due to the long-term treatment with sorafenib for 34 days. Yet in Figure 5C, tumors were treated for a shorter period. However, our tumor model still showed a relative resistance to sorafenib compared with the wild-type RENCA tumors (Figure 4G–I).

### 3.4. Activation of c-Met and AXL Contributes to Sorafenib Resistance in RCC

**Figure 6:**
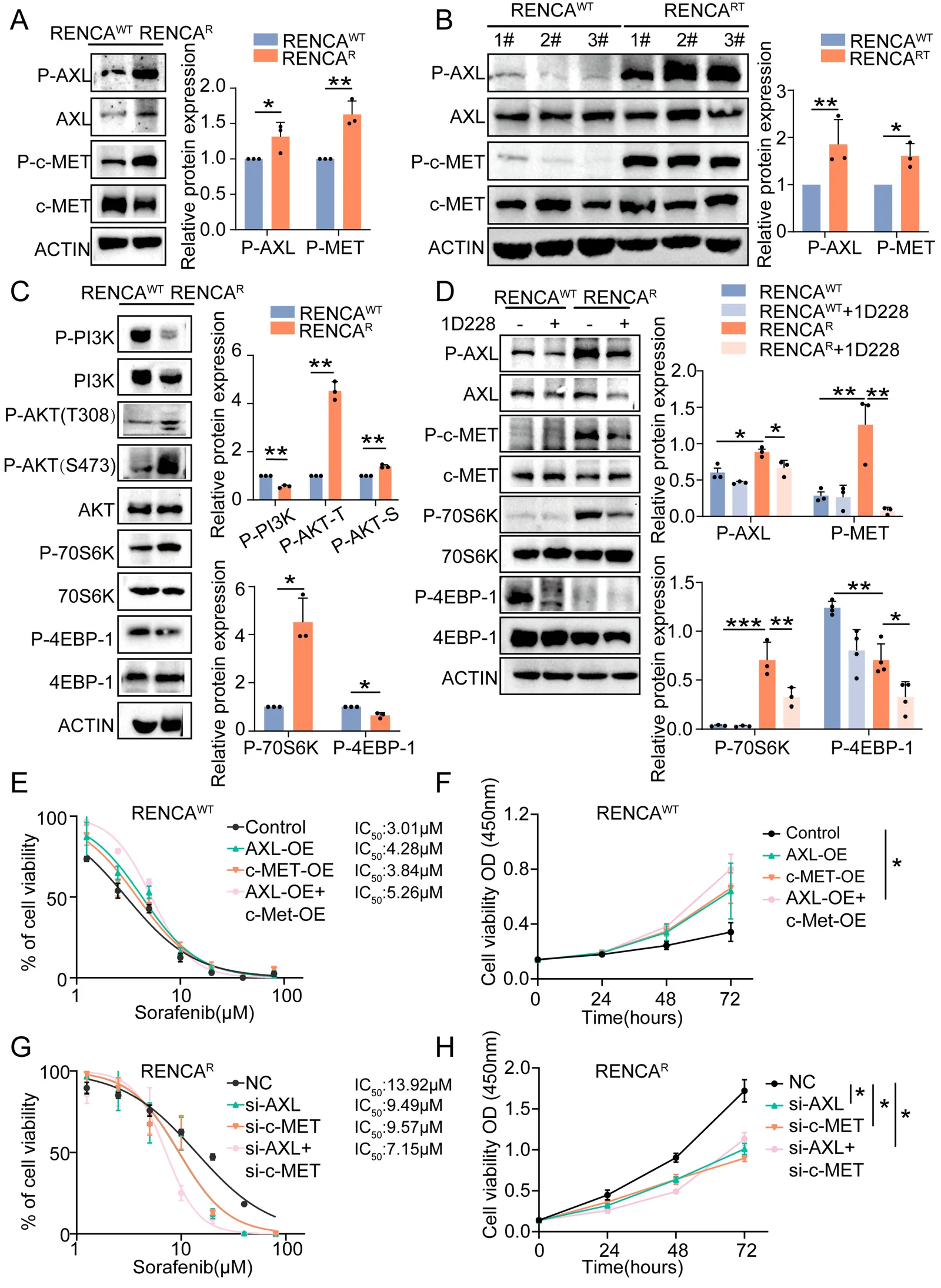
c-Met and AXL activation contributes to sorafenib resistance in RCC cells and tumor tissues. (**A**) Immunoblotting bands and relative quantification of AXL and c-Met activation in RENCA^WT^ and RENCA^R^ cells. (**B**) Comparison and relative quantification of AXL and MET pathway activation levels in RENCA^WT^ and RENCA^RT^ tumor tissues. *n* = 3. (**C**) Phosphorylation levels of key signaling proteins (PI3K, AKT pT308/pS473, 70S6K, 4EBP1) in RENCA^WT^ and RENCA^R^ cells. (**D**) Changes in phosphorylation levels of AXL/c-Met and mTOR pathway signaling in RENCA^WT^ and RENCA^R^ cells. Data for (**A**–**C**) were analyzed by *t*-test, and data for (**D**) were analyzed by one-way ANOVA, *n* = 3. *, *p* < 0.05; **, *p* < 0.01; and ***, *p* < 0.001. (**E**) Cell viability inhibition curves of RENCA^WT^ cells stably transfected with empty vector control, AXL overexpression, c-Met overexpression, and combined AXL/c-Met overexpression, following treatment with graded concentrations of sorafenib for 48 h. The corresponding *IC*_50_ values were 3.006, 4.280, 3.836, and 5.255 μM, respectively. *n* = 3. (**F**) Time-course proliferation curves of control, AXL-OE, c-Met-OE, and combined AXL/c-Met-OE RENCA^WT^ cells cultured in the presence of 5 μM sorafenib for 0, 24, 48, and 72 h. The data were obtained as determined by the CCK-8 assay. *n* = 3. (**G**) Cell viability inhibition curves of RENCA^R^ cells transfected with negative control, si-AXL, si-c-Met, and combined si-AXLc-MetT, following treatment with graded concentrations of sorafenib for 48 h. The corresponding *IC*_50_ values were 13.92, 9.485, 9.572, and 7.146 μM, respectively. *n* = 3. (**H**) Time-course proliferation curves of negative control, si-AXL, si-c-Met, and combined si-AXL/c-Met RENCA^R^ cells cultured in the presence of 5 μM sorafenib for 0, 24, 48, and 72 h. The data were obtained as determined by the CCK-8 assay. *n* = 3 Data are presented as mean ± SD. Statistical analysis was performed using two-way ANOVA. * indicates statistically significant differences between groups at *p* < 0.05.

The previous data demonstrated that 1D228 may overcome sorafenib resistance in RCC; however, the underlying mechanisms are not clear. In our early study, 1D228 selectively targets tyrosine kinases c-Met and AXL, which are involved in cancer progression. Thus, we hypothesize that the suppression effect in RENCA^R^ cells and the RENCA^RT^ tumors’ outcome was due to the inhibition of c-Met and AXL by 1D228 in the combination therapy. To validate this point, Western blotting was performed to test the activation of c-Met and AXL protein in both RENCA^WT^ and RENCA^R^ cells. Indeed, the phosphorylation level of c-Met and AXL was significantly increased in RENCA^R^ cells, indicating the activation of these signals (Figure 6A). Similarly, these two kinases were activated in RENCA^RT^ tumors as well (Figure 6B). These results suggested that c-Met and AXL activation is associated with sorafenib resistance in RCC.

To further study the mechanisms that are involved in sorafenib resistance in RCC, we did RNA-seq for RENCA^R^ cells and RENCA^RT^ tumors. Interestingly, the PI3K-AKT signaling pathway was clustered in both cell and tumor models, indicating that PI3K-AKT may be involved in sorafenib resistance (Figure S3). To verify this, we analyzed PI3K and AKT protein in RENCA^WT^ and RENCA^R^ cells. Interestingly, there was a mild reduction in the phosphorylation of PI3K; however, the phosphorylation of AKT at both threonine sites and serine sites was greatly increased in RENCA^R^ cells, indicating a strong activation of AKT by other mechanisms (Figure 6C). Since PI3K/AKT also regulates mTOR signaling, we examined the downstream signaling. Surprisingly, the two targets of mTOR 70S6K and 4E-BP-1 showed opposite changes. The phosphorylation of 70S6K was increased, while the phosphorylation of 4E-BP-1 was decreased. As 70S6K controls the efficiency of protein synthesis and 4E-BP-1 determines the initiation of protein translation, the controversial changes indicated a feedback mechanism in keeping resistant-cell survival when the protein synthesis was inhibited (Figure 6C). These results suggested that the decrease in p-4E-BP-1 might be due to the inhibition of mTOR signaling, and the increase in p-70S6K might be due to other signaling. To further investigate the signaling changes under the treatment of 1D228, RENCA^WT^ and RENCA^R^ cells were treated with the vehicle or 1D228. Consistently, p-AXL and p-c-Met were increased in RENCA^R^ cells and suppressed by 1D228, while there were hardly any changes in these two kinases in RENCA^WT^ cells, indicating 1D228 mainly targeted p-AXL and p-c-Met in resistant RCC cells (Figure 6D). Interestingly, p-70SK showed the same trend as p-AXL and p-c-Met, however, p-4E-BP-1 was blocked in both RENCA^WT^ and RENCA^R^ cells under 1D228 treatment, indicating an inhibition effect of 1D228 on 4E-BP-1 activation (Figure 6D).

To further validate the involvement of c-Met and AXL in sorafenib resistance, we did gain or loss of function studies in RENCA^WT^ or RENCA^R^ cells respectively. In RENCA^WT^ cells, overexpression of AXL and c-Met increased the *IC*_50_ value from 3.01 μM to 5.26 μM, which is a 1.75-fold elevation. The overexpression of these two genes also significantly promoted cell proliferation (Figure 6E,F). However, silencing c-Met and AXL in RENCA^R^ resulted in a decrease in *IC*_50_ value from 13.92 μM to 7.15 μM, which is a 1.95-fold reduction. Knocking down c-Met and AXL largely inhibited cell proliferation (Figure 6G,H). These data suggest that c-Met and AXL contribute to sorafenib resistance.

Since the combination of 1D228 and sorafenib targets multiple kinases, which might induce adverse effects by systemic drug administration, we examined the body weight, the morphology of main organs, and blood samples from the mice under the treatment of sorafenib, 1D228, and combination treatment. Notably, there were no body weight changes (Figure S4A). In the blood chemistry tests, liver function parameters including AST, ALP and TBA were not changed significantly. Although combination treatment induced ALT elevation, the average value (68.54 U/L) falls within the normal range (24–80 U/L). The same as with TP value, the combination group showed an average TP level of 48.02 g/L, which is within the normal range of 49–58 g/L (Figure S4B–F, Table S1). The kidney function parameters of CRE, URE, and UA did not show any significant changes (Figure S4H–J). Other blood parameters such as WBC, RBC, and hemoglobin did not show any changes. Combination therapy induced a decrease in platelet number, but the average value in the treated group falls in the normal range of 700–1100 × 10^9^/L (Figure S4J–M, Table S1). Additionally, H&E staining demonstrated no significant changes in organ morphology in the heart, liver, spleen, lung or kidney (Figure S4N). These data demonstrate that either 1D228 or combination treatment did not cause acute toxicity in mice.

To generalize our findings, we repeated these experiments with human cell models. It is reported that the human OSRC-2 cell line is resistant to sorafenib relative to other human RCC cells [53]. We also screened multiple human RCC cell lines by *IC*_50_ tests and found that OSRC-2 is relatively resistant to sorafenib compared with other cell lines (Figure S5A). Western blot analysis showed that OSRC-2 expressed much higher c-Met and AXL than 786-O (Figure S5B). Thus, we chose OSRC-2 and 786-O for subsequent experiments. In the CCK-8 assay, 786-O cells exhibited a similar trend to RENCA^WT^ cells, in that 1D228 did not show inhibition of cell viability, and the combination of 1D228 and sorafenib did not show further inhibition either. On the contrary, combination treatment demonstrated an enhanced inhibition on OSRC-2 cells (Figure S5C,D). Similarly, the cell scratch assay demonstrated that sorafenib could largely inhibit cell migration, while OSRC-2 cells did not respond to sorafenib (Figure S5E). When we introduced 1D228 treatment to these cells in the migration test, 1D228 did not cause any inhibition in 786-O cells but dramatically reduced the cell migration in OSRC-2 cells (Figure S5F,G). These results validated our findings in the murine cell line RENCA, which indicates that c-Met and AXL upregulation contribute to sorafenib resistance in RCC.

Together, these data demonstrated that AXL and c-Met are associated with sorafenib resistance, and 1D228 could resensitize the resistance by targeting p-AXL and p-c-Met, as well as 4E-BP-1 involved in protein translation initiation.

**Figure 7:**
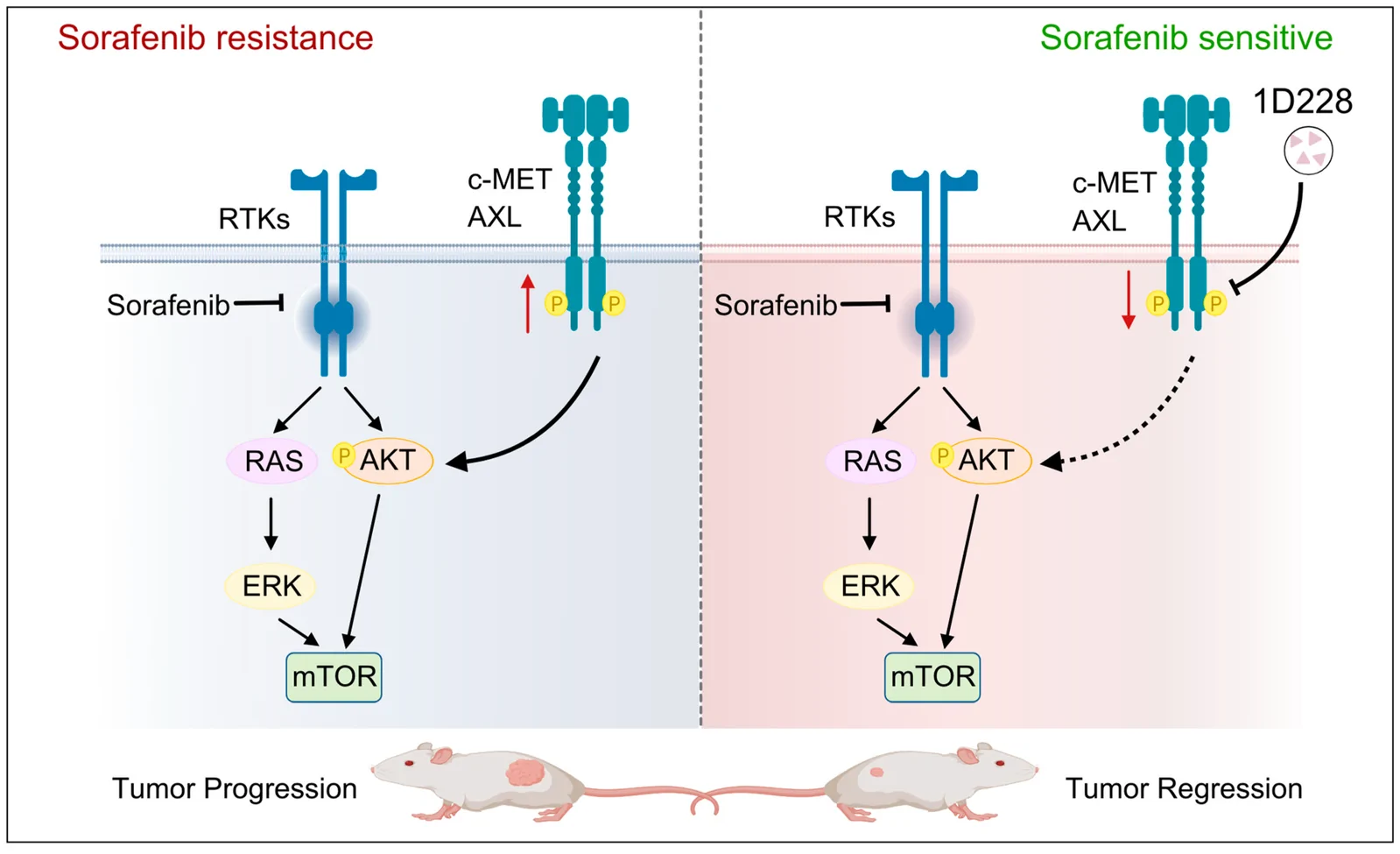
Targeted inhibition of the c-Met/AXL Axis by 1D228 overcomes sorafenib resistance in tumors. In RCC, sorafenib inhibited several receptor tyrosine kinases. However, after several treatment cycles, RCC develops the hyperactivation of c-Met and AXL signaling, which circumvents sorafenib suppression and results in tumor survival. When adding 1D228 to sorafenib treatment, c-Met and AXL-dependent tumor growth is blocked, and the tumor sensitivity is restored. Solid arrows indicate the activation of downstream signaling, where the dash arrows indicate weekend activation ability.

In summary, as illustrated in Figure 7, under the treatment of sorafenib, resistant RCC survives from the activation of c-Met and AXL, which contributes to the stimulation of AKT signaling, as well as part of the mTOR downstream signals. However, the combination of sorafenib and 1D228 simultaneously suppresses sorafenib-targeted kinases, c-Met, and AXL, leading to a broad inhibition on RCC, which partially resensitizes RCC resistance to sorafenib.

## 4. Discussion

Metastatic renal cell carcinoma is usually treated with TKIs, which benefits RCC patients in the form of prolonged survival. However, acquired resistance remains challenging in clinical practice. Sorafenib, a widely used TKI for hepatocellular carcinoma and renal cell carcinoma, is still recommended for anti-cancer therapy, but the same issue regarding acquired resistance has not been resolved. Studies on sorafenib resistance suggested multiple strategies to overcome tumor refractoriness, for example, combination with other TKIs to suppress the activation of feedback loop-induced kinases’ activities. C-Met is known to induce drug resistance, and tepotinib combined with sorafenib showed better inhibition on sorafenib-resistant tumors. But compared with cabozantinib, tepotinib is less effective as cabozantinib blocks both c-Met and AXL. Therefore, cabozantinib has been recommended as the second-line therapy for renal cell carcinoma. However, cabozantinib exhibited significant adverse effects, which undermined its benefits from the treatment. Thus, a more specific TKI drug with less toxicity is to be explored to meet the medical needs of sorafenib-resistant RCC patients.

In this study, we developed a sorafenib-resistant RCC cell line and a resistant tumor model. Based on these models, we tested our compound 1D228, which mainly targets c-Met and AXL. In the cell model, 1D228 exhibited no suppressive effects on cell proliferation, but induced apoptosis slightly. Interestingly, when combined with sorafenib, 1D228 showed no suppression on cell proliferation in WT cells but showed significant suppression in resistant cells. These data suggest that 1D228 does not have an effect on WT cells as they are not c-Met- and AXL-dependent. Similarly, in the in vivo models, a low dose of 1D228 at 2 mg/kg did not produce much inhibition on WT tumor growth either in monotherapy or combination therapy with 60 mg/kg sorafenib. But in the resistant tumor, the combination therapy showed strong inhibition on tumor growth, as well as antiangiogenic potential, which might be due to the dual targeting of AXL and NTRK. Further investigation by RNA-seq in WT and resistant RCC revealed that PI3K/AKT signaling was clustered in both cell models and tumor models, suggesting the cross talk between c-Met and AXL signaling. However, when examined, the mTOR downstream signaling showed opposite changes in 70S6K and 4E-BP-1. The increased p-70S6K and decreased p-4E-BP-1 levels suggest enhanced protein synthesis due to weakened translation initiation. However, under 1D228 treatment, the phosphorylation of c-Met, AXL, 70S6K, and 4E-BP-1 was largely blocked, demonstrating the potential of 1D228 in suppressing all the survival signaling.

## 5. Conclusions

In summary, our data demonstrated that the hyperactivation of c-Met and AXL is associated with sorafenib resistance in RCC. Our compound 1D228 may overcome sorafenib resistance by targeting both c-Met and AXL. Given that 1D228 has more potency than tepotinib on other types of cancers, which was validated in our previous study, these preclinical data suggest that 1D228 may be a promising candidate for further study in sorafenib-resistant RCC characterized by c-Met and AXL activation.

However, there are still limitations of the study. This study’s tests focused on one mouse RCC cell line for the in vivo study, and human cell lines and patient-derived-xenograft/orthotopic tumor models are more relevant to clinical situations. The comparison of cabozantinib or tepotinib vs. 1D228 in the resistant models is worth performing in the next study, which would strengthen the conclusion of the advantages of 1D228. The pharmacokinetic study and the extensive toxicity and tolerable studies would also be helpful to develop 1D228 as a therapeutic drug for cancer therapy, which needs further investigation.

## Data Availability

The datasets used and/or analyzed during the current study are available from the corresponding author on reasonable request. The raw data and original images from the present study are included in the Figshare database (not yet available). Further inquiries can be directed to the corresponding authors.

## Supplementary Materials

The following supporting information can be downloaded at: https://www.mdpi.com/article/10.3390/cells15161450/s1. Figure S1. Sorafenib or 1D228 treatment in RENCA sensitive or resistant cell lines; Figure S2. H&E staining of organs collected from mice treated with 1D228; Figure S3. Pathway analysis in RENCA^R^ cells and RENCA^RT^ tumors; Figure S4. Toxicity assessment of the combination therapy in mice; Figure S5. Combination of 1D228 and sorafenib suppressed sorafenib resistant human OSRC-2 cell proliferation and migration; Table S1. Blood Chemistry Panel.
