# Supplementary material for: 1D228 Attenuates Sorafenib Resistance in Renal Cell Carcinoma Models by Dual Targeting c-Met and AXL
Source: Cells. 2026 Aug 12;15(16):1450. doi: 10.3390/cells15161450 (PMC13510577; doi:10.3390/cells15161450)
Supplement: Supplementary file 1 [file cells-15-01450-s001.zip › cells-4363776-supplementary.pdf]

Supplemental Information

Supplemental methods

RNA sequencing analysis

In this study, two sets of mouse transcriptome sequencing experiments were conducted, comprising six mouse cell samples (three $RENCA^{WT}$ and three $RENCA^{R}$ cell samples) and eight mouse tumor samples (four $RENCA^{WT}$ and four $RENCA^{RT}$ tumor samples). The sequencing was done by Igenecode, Beijing, China. Two main normalization algorithms were employed: the primary quantitative metric was FPKM, which corrects for the confounding factors of sequencing depth and gene length; raw read counts were retained as auxiliary data for subsequent differential expression analysis. The experimental design considered the presence or absence of biological replicates. Differential gene detection was performed using four algorithms: DESeq2, EBSeq, NOIseq, and PoissonDis, each with fixed filtering thresholds (fold change + statistical test), as described below:

DESeq2: Fold Change ⩾ 2.00 and Adjusted P-value ⩽ 0.05
EBSeq: Fold Change ⩾ 2.00 and Posterior Probability of being Equivalent Expression (PPEE) ⩽ 0.05
NOIseq: Fold Change ⩾ 2.00 and Probability ⩾ 0.8
PoissonDis: Fold Change ⩾ 2.00 and FDR ⩽ 0.001
P-value: An indicator of statistical significance from the original hypothesis test, uncorrected.
Padj/FDR: The P-value after multiple testing correction (core reporting metric); smaller values indicate more significant differential gene expression. Gene expression levels, log2FoldChange, and up/downregulation results are also presented.
For the six mouse cell samples, the average data output per sample was 7.05 Gb. The average alignment rate of sequencing reads to the mouse reference genome was 98.39%, and an average of 13,849 expressed genes were detected per sample. The FPKM normalization pipeline for these samples was as follows: clean reads were aligned to the reference gene sequences (supplemented with novel transcripts) using Bowtie2. Unlike the reference genome, the reference gene sequences contain the base sequences of all transcripts. Subsequently, RSEM (version 4) was used to calculate gene and transcript expression levels, followed by downstream analyses.

For the eight mouse tumor samples, the average data output per sample was 8.52 Gb, and the average alignment rate to the reference genome was 97.96%. On average, 1,890 differentially expressed genes were detected per differential analysis group. The FPKM normalization pipeline for these samples was as follows: sequencing reads were first aligned to the reference genome using HISAT2, and then the StringTie + Ballgown software package was used to calculate the FPKM value for each gene/transcript.

The raw data is uploaded to NIH database submission. SRA ID: SRP717053.

Supplemental figures

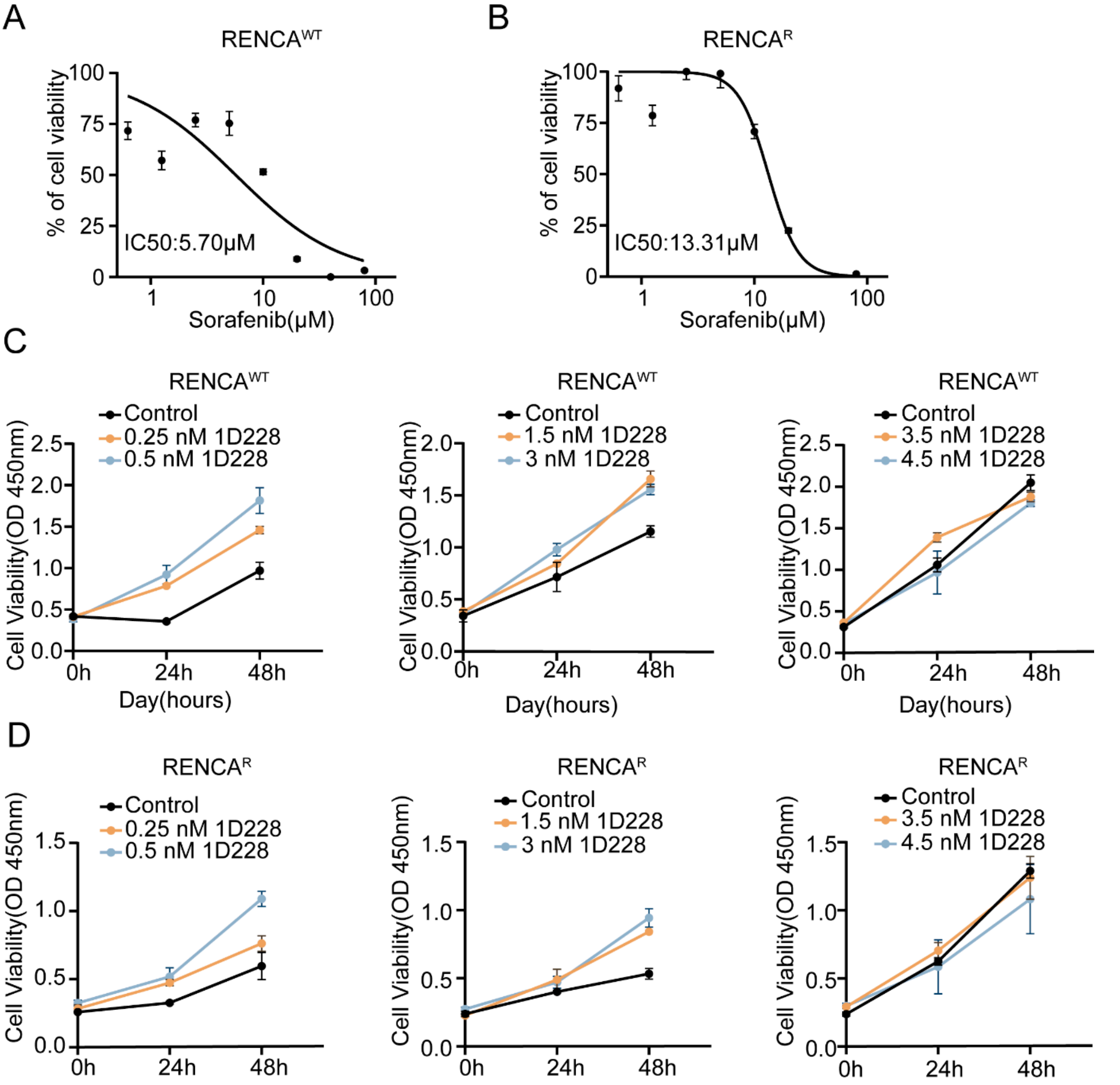

Figure S1. Sorafenib or 1D228 treatment in RENCA sensitive or resistant cell lines. A-B) The IC50 of RENCA^{R} assessed at passage 12 days after sorafenib withdrawal. Dose-response curves showing the percentage of cell viability of RENCA^{WT} (A) and RENCA^{R} (B) cells treated with increasing concentrations of Sorafenib (1–80 μM) for 48 hours. The IC_{50} values are indicated within the graphs. RENCA^{WT}: 5.704 μM, RENCA^{R}: 13.31 μM. n=3. **C, D)** Time-course (0, 24, 48 h) of cell viability (OD 450 nm) in RENCA^{WT} (C) and RENCA^{R} (D) cells

treated with control or varying concentrations of 1D228 (0.25 nM, 0.5 nM, 1.5 nM, 3 nM, 3.5 nM, 4.5 nM). n=3. Data is presented as mean ± SD.

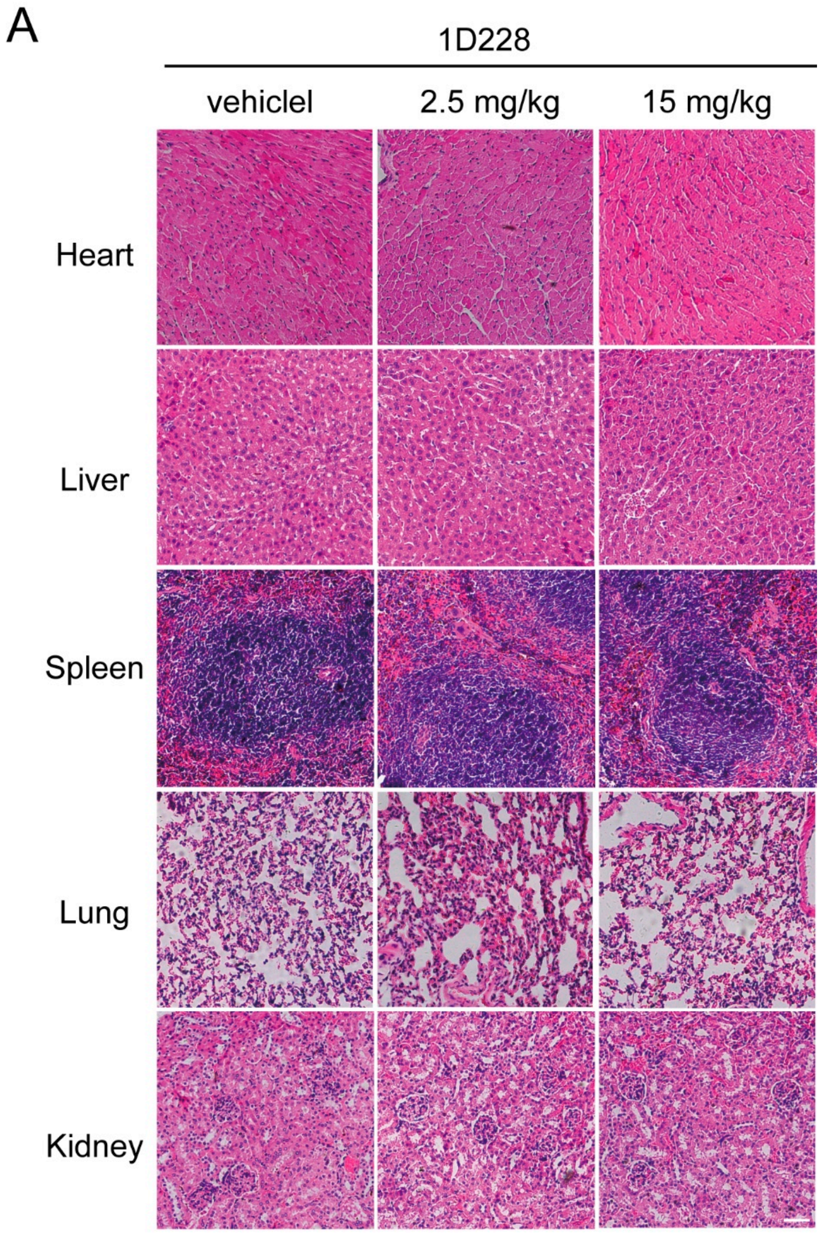

Figure S2. H&E staining of organs collected from mice treated with 1D228. A) Representative H&E-stained tissue sections of major organs (heart, liver, spleen, lung, and kidney) from mice treated with vehicle, 2.5 mg/kg 1D228, and 15 mg/kg 1D228. No obvious pathological lesions or morphological abnormalities were observed in the 1D228-treated groups compared to the control. n=3-4. The total number of mice in the study was 10. Bar: 50 μm.

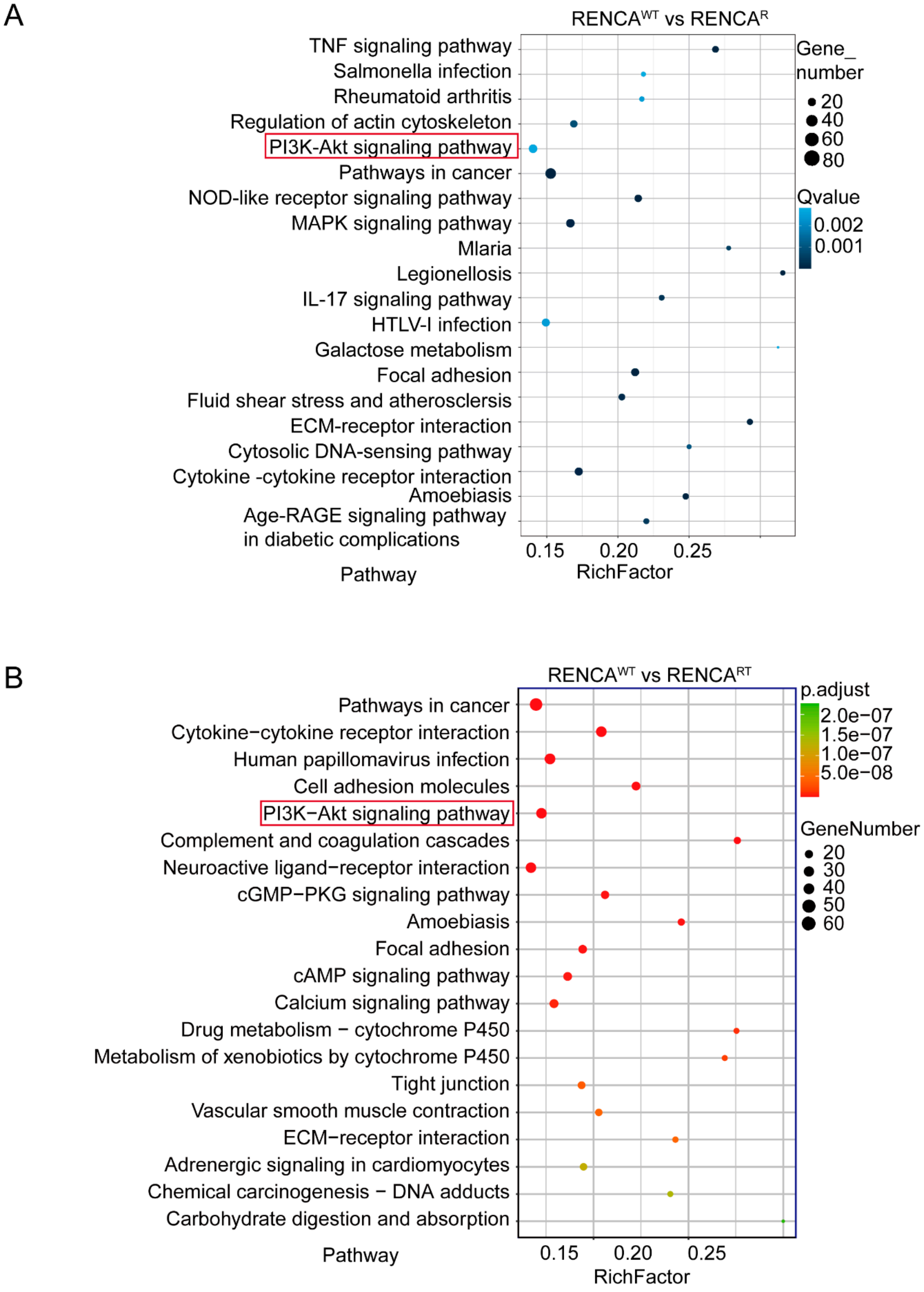

Figure S3. Pathway analysis in RENCA^{R} cells and RENCA^{RT} tumors. A) The top 20 clustered pathways in sorafenib resistant cells. n=3 **B)** The top 20 clustered pathways in sorafenib resistant tumors. n=4. The total number of mice in the study was 8.

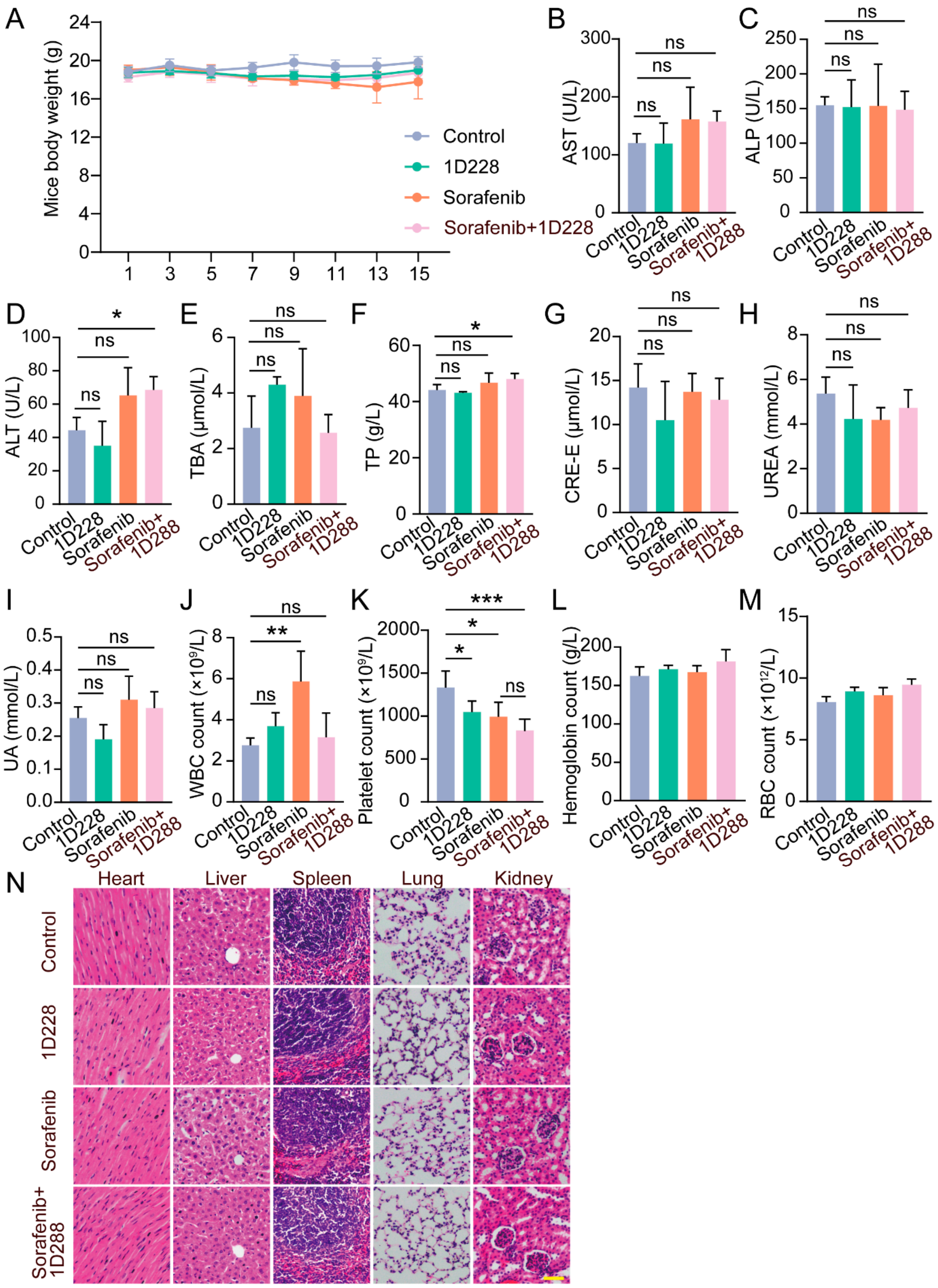

Figure S4. Toxicity assessment of the combination therapy in mice.

A) Mice body weights recorded every other day in the Control, 1D228, Sorafenib, and Combination treatment groups. **B–M)** Serum biochemistry and hematological parameters

assessed at the experimental endpoint, including AST (B), ALP (C), ALT (D), TBA (E), TP (F), CRE-E (G), URE (H), UA (I), WBC count (J), Platelet count (K), Hemoglobin count (L), and RBC count (M). **N)** H&E staining of major organs (heart, liver, spleen, lung, and kidney) from mice in each treatment group. n=4-5. The total number of mice in the study was 18. Bar: 50 μm. Data are presented as mean ± SD. Statistical significance was determined by one-way ANOVA. * $P < 0.05$, ** $P < 0.01$, *** $P < 0.001$; ns, not significant. AST: Aspartate Aminotransferase; ALT: Alanine Aminotransferase; ALP: Alkaline Phosphatase; TBA: Total Bile Acid; TP: Total Protein; CRE-E: Creatinine; URE: Urea; UA: Uric Acid; WBC: White Blood Cell; RBC: Red Blood Cell; Hemoglobin (Hb) Platelet (PLT)

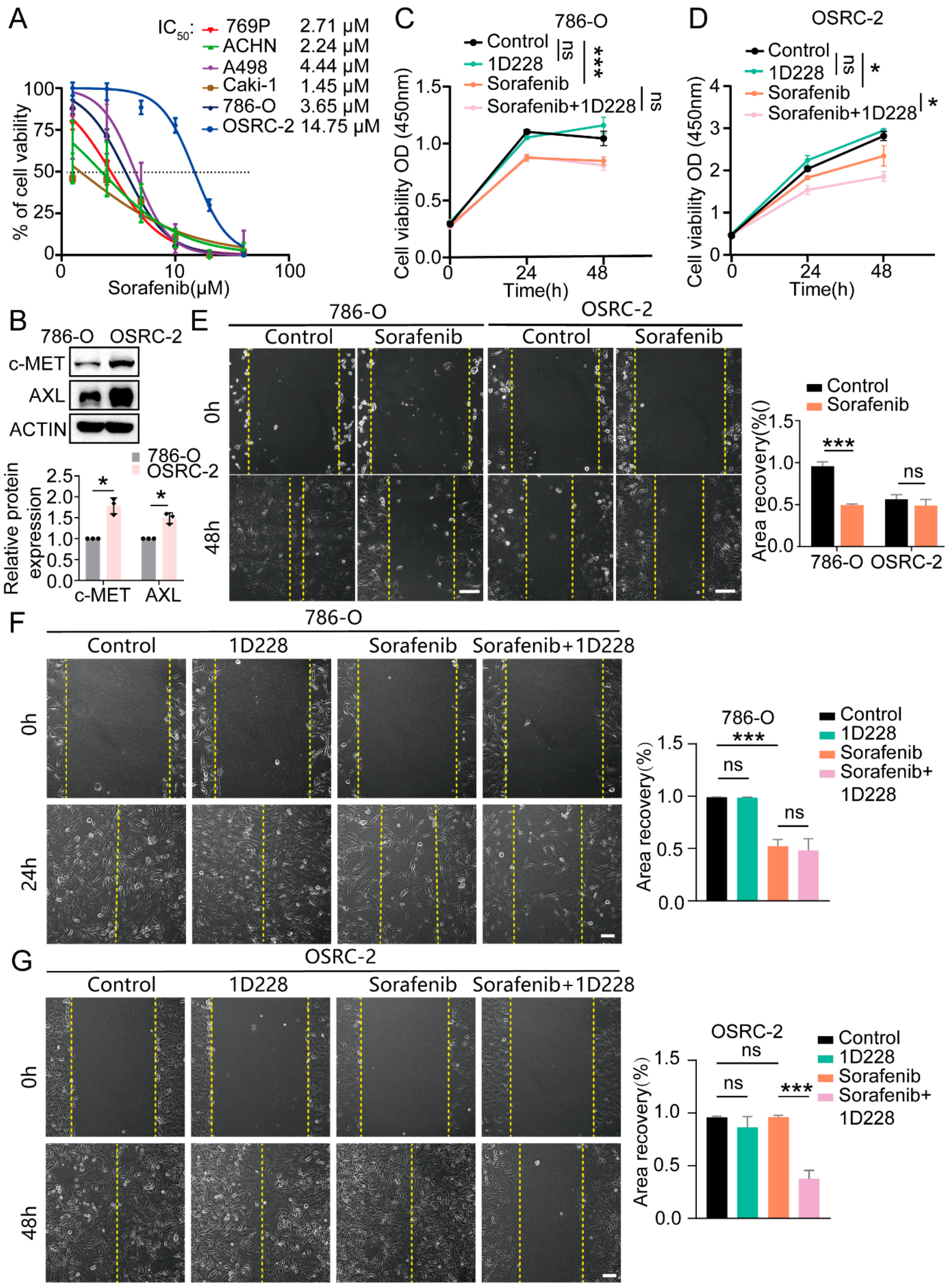

Figure S5. Combination of 1D228 and sorafenib suppressed sorafenib resistant human OSRC-2 cell proliferation and migration. A) A CCK-8 cell viability assay was performed to evaluate the cell viability of six clear cell Renal Cell Carcinoma (ccRCC) cell lines following

treatment with various concentrations of sorafenib for 48 hours. n=3. **B)** Western blot detection of c-MET and AXL protein expression in 786-O and OSRC-2 ccRCC cells. β-ACTIN served as loading control. **C-D)** Proliferation of 786-O (B) and OSRC-2 (C) cells following treatment with control, 1D228 (10 nM), sorafenib (2 μM) or 1D228 in combination with sorafenib. n= 3. **E)** Wound healing assay was performed to assess the migratory capacity of 786-O and OSRC-2 cells at the same sorafenib concentration (2 μM), with quantitative analysis of wound closure rate for each group, Control, sorafenib. n=3. bar: 50 μm. **F-G)** Scratch assay of 786-O (F) and OSRC-2 (G) cells migration in different groups. The percentage of wound closure was quantified for each group. Control (DMSO). Sorafenib: 2 μM. 1D228: 40nM. Combination. n=3. bar: 50 μm. Statistical analysis was performed using Student's t-test for data in A, one-way ANOVA for data in C-D, and two-way ANOVA for data in F-G,. All data are presented as mean ± SD. ns, not significant; *, $P<0.05$; ***, $P<0.001$.

Supplemental Table S1. Blood chemistry panel

Groups / Parameters	Control	1D228	Sorafenib	Sorafenib+1D228	Normal range
AST (U/L)<br>Mean±SD<br>Raw data	120.58±15.86<br>(98.1~139.5)	84.39±154.76<br>(72.1~154.9)	161.70±55.28<br>(120.7~242.8)	157.36±18.24<br>(143.8~189.4)	120~220
ALP (U/L)<br>Mean±SD<br>Raw data	154.78±12.12<br>(136.8~164.10)	152.2±39.11<br>(102~185)	153.88±60.24<br>(70.5~206)	148.20±26.71<br>(102.6~171)	140~240
ALT (U/L)<br>Mean±SD<br>Raw data	44.36±7.71<br>(33.5~50.5)	35.00±14.30<br>(13.2~45)	65.23±16.68<br>(46.1~81.3)	68.54±7.97<br>(62.1~80.7)	24~80
TBA (μmol/L)<br>Mean±SD<br>Raw data	2.75±1.14<br>(1.35~4.53)	4.31±0.28<br>(3.94~4.54)	3.90±1.70<br>(2.42~5.44)	2.57±0.66<br>(1.8~3.32)	1.0~9.8
TP (g/L)<br>Mean±SD<br>Raw data	44.12±1.93<br>(40.8~45)	43.13±0.38<br>(42.8~43.5)	46.73±3.46<br>(43.8~51.5)	48.02±7.94<br>(46.2~51)	49~58
CRE-E (μmol/L)<br>Mean±SD<br>Raw data	14.21±2.68<br>(10.9~16.97)	10.49±4.43<br>(4.28~14.76)	13.73±2.09<br>(10.9~15.86)	12.83±2.43<br>(10.7 ~15.86)	14~29
UREA (mmol/L)<br>Mean±SD<br>Raw data	5.3±0.74<br>(4.47~6.46)	4.23±1.53<br>(2.12~5.77)	4.19±0.55<br>(3.47~4.79)	4.72±0.81<br>(4.11~6.07)	4.1~8.4
UA (mmol/L)<br>Mean±SD<br>Raw data	0.25±0.03<br>(0.2115~0.29)	0.19±0.04<br>(0.135~0.24)	0.31±0.07<br>(0.21~0.38)	0.29±0.05<br>(0.206~0.33)	0.234~0.381
WBC ($\times10^9$/L)<br>Mean±SD<br>Raw data	2.76±0.36<br>(2.22~3.18)	3.69±0.65<br>(2.9~4.5)	5.87±1.48<br>(4.38~7.9)	3.15±1.18<br>(1.86~4.82)	2.5~12.5
Platelet ($\times10^9$/L)<br>Mean±SD<br>Raw data	1332.00±194.52<br>(1129~1584)	1047.5±128.3<br>(897~1207)	992±168.73<br>(748~1134)	832.2±103.34<br>(689~948)	700~1100
Hemoglobin (g/L)<br>Mean±SD<br>Raw data	162.4±11.78<br>(165~179)	171.25±4.99<br>(166~178)	167.5±8.39<br>(156~176)	181.4±15.37<br>(158~197)	120~160
RBC ($\times10^{12}$/L)<br>Mean±SD<br>Raw data	8.06±0.45<br>(7.53~8.51)	8.93±0.33<br>(8.54~9.35)	8.6±0.62<br>(7.95~9.45)	9.45±0.47<br>(8.7~9.87)	7.5~10

AST: Aspartate Aminotransferase; ALT: Alanine Aminotransferase; ALP: Alkaline Phosphatase; TBA: Total Bile Acid; TP: Total Protein; CRE-E: Creatinine; URE: Urea; UA: Uric Acid; WBC: White Blood Cell; RBC: Red Blood Cell; Hemoglobin (Hb) Platelet (PLT)
